# PROTOCOL: Mapping the scientific knowledge and approaches to defining and measuring hate crime, hate speech, and hate incidents

**DOI:** 10.1002/cl2.1228

**Published:** 2022-04-18

**Authors:** Matteo Vergani, Barbara Perry, Joshua Freilich, Steven Chermak, Ryan Scrivens, Rouven Link

**Affiliations:** ^1^ Alfred Deakin Institute for Citizenship and Globalisation Deakin University Burwood Victoria Australia; ^2^ Centre on Hate, Bias and Extremism Ontario Tech University Oshawa Ontario Canada; ^3^ John Jay College of Criminal Justice (CUNY) City University of New York New York New York USA; ^4^ School of Criminal Justice Michigan State University East Lansing Michigan USA

## Abstract

The overallaim of the review is to map the definitions and measurement tools used to capture the whole spectrum of hate motivated behaviors, including hate crime, hate speech and hate incidents. This will benefit the field of hate studies by providing a baseline that can inform the building of cumulative knowledge and comparative research. The first review objective is to map definitions of hate crime, hate incidents, hate speech, and surrogate terms. Specific research questions underpinning this objective are: (a) How are hate crimes, hate speech and hate incidents defined in the academic, legal, policy, and programming literature?; (b) What are the concepts, parameters and criteria that qualify a behavior as being hate crime, hate incident or hate speech?; and (c) What are the most common concepts, parameters and criteria found across definitions? What are the differences between definitions and the elements they contain? The second review objective is to map the tools used to measure the prevalence of hate crime, hate incidents, hate speech, and surrogate terms. Specific research questions underpinning this objective are: (a) How are definitions operationalised to measure hate crimes, hate speech, and hate incidents?; and (b) How valid and reliable are these measures?

## 1 BACKGROUND

### 1.1 The problem, condition, or issue

There is limited international consensus for how to define behaviors motivated by hate or containing a hate element, which include hate speech, hate incidents and hate crime (Schweppe, 2021). For some, a hate crime is any criminal behavior motivated by hate against protected identities or minority communities (Office for Democratic Institutions and Human Rights [ODIHR], 2009). For others, hate crime captures all malicious behaviors motivated by hate, ranging from behavior regulated by criminal law, by civil law, or not regulated at all (Chakraborti & Garland, 2015; Hardy, 2019). This definition overlaps with what practitioners often define as hate incidents, that is, all malicious behaviors motivated by hate that fall below the threshold of criminality (Anti‐Defamation League, 2019). Some use the term “hate incident” to capture all malicious behavior motivated by bias, including both criminal and noncriminal acts (Sadique et al., 2018). The definitions of hate crime and hate incidents overlap with the concept of hate speech, which includes verbal or non‐verbal manifestations of hatred, such as gestures, words or symbols like cross‐burnings, bestial depictions of members of minorities, hate symbols, among others (Strossen, 2018). Some of these behaviors—for example, incitement to hatred, Holocaust denial—might be regulated by criminal law in certain jurisdictions (thus overlapping with some definitions of hate crime), by civil law, or not regulated at all (thus overlapping with some definitions of hate incidents). Determining whether a crime is motivated by hate is a well‐known challenge in the literature, which led to the adoption of different definitional models: the “animus model” and the “discriminatory selection model” (Lawrence, 1999). The animus model requires that the hate element (i.e., a form of bias, prejudice or hostility) is present and visible in the crime. For example, the offender might be seen as yelling a racial slur while attacking the victim. Conversely, under the discriminatory selection model, a crime is defined as hate motivated by reason of the victim's characteristics and perceived identity. For example, selecting a victim from a minority group is sufficient to define a crime as a hate crime (Lawrence, 1999).

Cross‐cultural research found that, at an international level, hate crime and hate speech statutes are strongly influenced by the different social, technical, historical and cultural contexts across nations (Sheppard et al., 2021). For example, Italy has banned the display of ideas and symbols of fascism with the Law 205/1993 known as Mancino law (Campani, 2016). In the German context, the legacy of the Holocaust is mainly responsible for the criminalization of public expressions of hate that could engender or promote violence to protected groups such as Holocaust denial and trivialization (Bleich, 2011; Kahn, 2005). On the other hand, in the United States freedom of speech is constitutionally protected and has been a central tenet of individual liberty that has prevented the country from passing stringent laws. But, some forms of speech are criminalized in the United States, such as speech that incites imminent threat of violence (Heyman, 2009). In Canada, the Parliament amended the Criminal Code in 1970, thus rendering hate propaganda as a punishable offence. These laws fall under sections 318‐320 of the Criminal Code. Four specific offences are listed as hate propaganda offences or hate crimes in the Criminal Code of Canada: advocating genocide, public incitement of hatred, wilful promotion of hatred and mischief motivated by hate in relation to religious property. In addition, subparagraph 718.2(a)(i) of the Criminal Code allows for increased penalties when sentencing any criminal offence (such as assault or mischief) where there is evidence that the offence was motivated by bias, prejudice or hatred toward a particular group as listed in the Criminal Code. These are also considered hate crimes.

The term “hate” is sometimes criticized by scholars and practitioners who find it ambiguous and used normatively to criminalize political opponents. For these reasons, some scholars and practitioners use surrogate terms to refer to the concepts of hate crime, hate speech and hate incidents. For example, in Australia, hate crimes are referred to as bias crimes in New South Wales, and as prejudice‐motivated crimes in Victoria. Moreover, hate crimes, hate speech and hate incidents are often captured using terms like racism, anti‐Semitism or homophobia (among others). In the literature, these community‐specific terms are used to capture attitudes and behaviors interchangeably. In some jurisdictions, there is a considerable overlap between the concept of hate crime and neighboring concepts like “extremism” and “terrorism.” For example, several European countries criminalize membership of extremist groups, and include these acts within their national concepts of hate crime (Perry, 2016). In the United States, domestic terrorism is often defined as hate crime (Taylor, 2019). Many events blur the line between hate crimes and terrorism, such as the Pittsburgh Synagogue shooting in 2018 and the Christchurch attack in 2019 (Vogel‐Scibilia, 2020).

Globally, various data sources are used to track trends of hate behaviors. They are mostly disconnected, are shaped by different legislations and use different terminology, criteria and definitions, and therefore are not comparable (often even within the same country). A large portion of information on the extent of hate crimes comes from law enforcement records. However, these registers are affected by numerous limitations, including law enforcement misclassifications, differences in reporting patterns by communities, scarce funding allocated to data collection and training of police officers recording the data, as well as differences between jurisdictions in legislation, thresholds for proof of bias motivation, data collection criteria, time periods considered in country reports, differences in visibility and acceptance of legislations (Sheppard et al., 2021). All these limitations hinder our ability to conduct comparative research on hate crime, hate speech and hate incidents. Although law enforcement hate crime data within certain countries might still provide valuable information on temporal trends, cross‐country comparison is methodologically impossible. Multinational organizations such as the OSCE's Office for Democratic Institutions and Human Rights and the EU Fundamental Rights Agency publish reports of hate crimes reported to police and prosecuted across multiple countries (e.g., ODIHR, 2009). However, their data is hindered by the limitation of their sources.

Victimization surveys are another official source of data about hate crime, although questions about the bias motivation of a crime are included only in a few countries (e.g., the United States, Canada, England, and Wales). These surveys are often used to assess the so‐called “dark figure” of hate crime, and to measure under‐reporting. However, they can be limited by methodological issues such as: being exclusively based on the victim's perception to determine the bias motivation; the perception of what constitutes a crime and what constitutes hate varies across demographic and community groups; language barriers among linguistically diverse respondents; limitations in the questions asked (e.g., missing categories of victim groups including age groups or target identities such as the homeless); and fail to record lethal incidents, public order offences, and hate crimes happening in certain locations (such as dormitories, military barracks, jails, penitentiaries, assisted care facilities) (Sheppard et al., 2021). The Crime Survey for England and Wales (CSEW) excludes so‐called “victimless” crimes, such as some public order offences where no direct victims are identified (which constitute about 50% of police recorded hate crime) and it does not survey people in multiple occupancy residences, such as student halls or retirement homes (M. Williams, 2021). Some organizations like the EU Fundamental Rights Agency run longitudinal cross‐jurisdictional surveys with minority communities that include a focus on hate crime, for example, the LGBT Rights (I and II), EU MIDIS I and II, Roma and Traveller Survey.

Other surveys conducted by government and nongovernment organizations (such as universities, research institutes and think tanks) can explore impacts and manifestations of hate crimes, hate incidents and hate speech (Benier et al., 2016; Walters et al., 2019). Examples are the Leicester Hate Crime Project and the All Wales Hate Crime Project (see M. L. Williams & Tregidga, 2014).

Community organizations and watch‐groups often collect data about hate crime, hate speech and hate incidents (both above and below the criminal thresholds) within their communities. Notable examples are Tell MAMA (measuring anti‐Muslim attacks in the United Kingdom), the Anti‐Defamation League (measuring anti‐Semitic attacks in the United States), and B'nai Brith (measuring anti‐Semitic incidents in Canada and other countries). Data collected by civil society organizations can be extremely valuable for research purposes and are often used in scholarly research (Green et al., 2001; Vergani et al., 2021). Some argue that watch groups might have a vested interest in inflating the perception of hate against the communities they represent (Kaplan, 1997). While acknowledging this critique, we believe that, in a context where hate crime data quality is often suboptimal because of all the limitations outlined above (Gerstenfeld & Grant, 2004; Saucier et al., 2006), community registers are a key source of data that can be used to compare to other sources (Mason et al., 2017).

Both on‐ and offline media are an important source of primary and secondary hate data. Web searches can be used to retrieve media coverage, law enforcement reports, and non‐profit reports to create a database of bias and hate crimes incidents, as in the case of ProPublica. On‐ and offline media can also be the vehicle of hate speech, and there is a growing scholarship focusing on creating effective automated detection tools to capture and measure it (see Poletto et al., 2021; Williams, 2021). Automatic detection of hate speech online is a direct observation that does not require an input from victims or witnesses, and it is a growing field with known problems of accuracy, validity, reliability, sensitivity and specificity of the tools, which can result in false negatives and false positives (M. Williams, 2021).

Other government agencies such as National Human Rights Institutions (NHRIs) often collect reports of hate incidents both above and below the criminal threshold. In countries where NHRIs are tasked with dealing with incidents regulated by civil law (e.g., in Australia), they are an important repository of hate incidents and hate speech reports, although they are often prevented by privacy regulations to share the data that they collect. Government statistical agencies are also important to mention, such as Canada's Statistics Canada and the Canadian Centre for Justice and Community Safety Statistics (CCJCSS).

The proliferation of language used to refer to similar concepts, the lack of definitional clarity and the limitations of measurement approaches pose real challenges to policy, practice and research focusing on tackling hate crime, hate speech and hate incidents. These challenges include problems in:

1) developing valid and reliable measurement tools to measure hate behaviors, which poses a barrier to:
  a. understanding the real magnitude and trends of hate behaviors;
  b. evaluating the impact of policy and programs across different jurisdictions and build an understanding of what works and what does not;
  c. building comparative and cumulative knowledge of the causes and solutions of hate behaviors;
2) developing legal standards, including:
  a. establishing standards for defining the hate element via mechanisms such as demonstrating the presence of a hate element, the hate motivation or the discriminatory selection of the victims;
  b. identifying the groups warranting protection;
  c. treating victims of hate equally across different jurisdictions (both between and within countries) and between groups;
  d. applying legal definition to real cases, and utilizing hate crime statutes for criminal prosecution, sentencing, post‐conviction and restorative justice;
  e. developing appropriate legislative structures (such as sentencing and offences);
3) raising awareness about hate among victims and target groups, among various professional groups including police officers, social workers, government officials, as well as the public;
4) developing effective policing and policies (including prosecutorial policies and judicial guidance) on hate crime, hate speech and hate incidents, to make it easier navigating the system of reporting hate, receiving support and the criminal justice system for victims of hate;
5) assessing empirically how and to what extent incidents like hate speech (including online hate speech) and hate incidents might constitute early warnings for hate crime and terrorism incidents.

This review will move this field of hate studies (i.e., studies on hate crime, hate speech, hate incidents and surrogate terms identifying specific forms of hate such as racism, anti‐Semitism and homophobia) toward more empirical rigor and theoretical clarity by mapping current and historical approaches to defining and measuring hate crime, hate incidents, hate speech and surrogate terms in Canada, North America, Europe, Australia and New Zealand.

### 1.2 Why it is important to do the review

Hate has been a persistent problem across human history (Sternberg, 2003), and it has become an ever more pressing issue in the wake of the COVID‐19 pandemic, which has sparked a wave of anti‐Asian, anti‐foreigner, anti‐Muslim and anti‐Semitic hate. The report by the Royal Commission of Inquiry into the 2019 Christchurch terrorist attack highlighted the importance of having comprehensive and effective hate crime legislation, of collecting better hate crime data, and of creating a workable approach to tackling hate speech, as means to prevent terrorist attacks (Veilleux‐Lepage et al., 2020). Despite the relevance of this policy area, the ability of states to create effective policies to address behaviors motivated by (or demonstrating) hate (i.e., hate crimes, hate incidents and hate speech) is constrained by a general lack of clarity of what constitutes hate, and how to measure it.

Stakeholders have been calling for a mapping of definitions and measurement tools of the whole spectrum of hate behaviors to map the current developments in policy, practice and research (Schweppe, 2021; Sheppard et al., 2021). This mapping will help government and nongovernment stakeholders in North America, Europe, Australia and New Zealand inform the next generation of policies, programs, and research, as well as advocacy for improving legislation. The reasons behind the choice of these jurisdictions is explained in the “Population” section.

This project's outcomes (i.e., the final report and other publications) will provide a comprehensive mapping of the current portfolio of definitions and measurement tools available in North America, Europe, Australia and New Zealand. This will support the critical appraisal of strengths and weaknesses of different country‐approaches, and the strategic planning and development of the next wave of research, policy and legislative efforts and shape the next generation of anti‐hate efforts globally.

### 1.3 How this review might inform or supplement what is already known in this area

Many scholars have discussed the problems associated with the lack of consistent definitions and measurement of hate crime, hate speech and hate incidents both within federal countries like the United States and across different countries in North America and Europe (see Schweppe, 2021; see also Sheppard et al., 2021). They outlined the main issues (as discussed in the first section of this protocol) and highlighted the tendency for researchers, policy makers and practitioners to work in silos, each developing their own definitions and measurement of hate crime with little (or no) dialogue across sectors (Chakraborti & Garland, 2015; Perry, 2016).

However, no study to date has systematically mapped the field of hate studies. This review will provide the first comprehensive mapping of the field by looking at:

1) the whole spectrum of hate behaviors above and below the criminal threshold (including hate crime, hate incidents and hate speech);
2) the components of definitions disaggregated by the behavior captured (whether a behavior is regulated by criminal law, civil law, or not regulated, whether it includes expressions of opinions or ideas), the hate motivation (whether it is captured by bias indicators, and if so what they are; whether the perception of the victim or a witness is considered as a bias indicator), the targets of hate (whether any identity or group can be target of hate, or only certain protected characteristics that capture marginalized groups, and if so, which ones they are);
3) different areas of scholarship and practice, including law and statutes, scholarship (differentiating between disciplines), practitioners (differentiating between different types of government and nongovernment organizations);
4) change across different geographical areas;
5) change over time;
6) different surrogate terms used to capture the concepts of hate crime, hate speech and hate incidents, including terms used in different jurisdictions (e.g., prejudice motivated crime) and community‐specific terms (e.g., anti‐Semitism);
7) different types of measurement tools, including third party reporting systems and victimization surveys.

The review will allow us to unpack strengths and weaknesses of definitions and their operationalization for measurement, and discuss legislation, program and policy gaps overall, which will allow us to draw conclusions relevant for North America, Europe, Australia and New Zealand.

We conducted a search of the literature using the following terms to identify existing reviews: hate crime* OR hate speech* OR hate incident*. Searches of the following locations did not identify any existing systematic reviews (completed or ongoing) on the specific topic proposed in this proposal (i.e., definitions and measurements of hate crime, hate incidents and hate speech):

- Campbell Collaboration
- Cochrane Collaboration
- PROSPERO registry
- Google Scholar

## 2 OBJECTIVES

The overall aim of the review is to map the definitions and measurement tools used to capture the whole spectrum of hate motivated behaviors, including hate crime, hate speech and hate incidents. This will benefit the field of hate studies by providing a baseline that can inform the building of cumulative knowledge and comparative research.

The first review objective is to map definitions of hate crime, hate incidents, hate speech, and surrogate terms. Specific research questions underpinning this objective are: (a) How are hate crimes, hate speech and hate incidents defined in the academic, legal, policy, and programming literature?; (b) What are the concepts, parameters and criteria that qualify a behavior as being hate crime, hate incident or hate speech?; and (c) What are the most common concepts, parameters and criteria found across definitions? What are the differences between definitions and the elements they contain?

The second review objective is to map the tools used to measure the prevalence of hate crime, hate incidents, hate speech, and surrogate terms. Specific research questions underpinning this objective are: (a) How are definitions operationalised to measure hate crimes, hate speech, and hate incidents?; and (b) How valid and reliable are these measures?

## 3 METHODOLOGY

### 3.1 Criteria considering studies for this review

Some criteria apply to both objectives (see “population,” “phenomenon of interest,” and “context” sub‐sections).

#### 3.1.1 Types of studies

For both objectives, this review will include the following types of documents published after 1990:

(1) academic literature in the social and psychological sciences (including criminology, sociology, political science, law and psychology, computer science and sub‐fields like regional studies, religious studies and peace and conflict studies);
(2) gray literature by watch‐groups and other governmental and nongovernmental organizations.

Additionally, this review will include current legislation, including hate crime, hate speech, and hate incidents found in federal and state statutes, including criminal and civil law statutes, and international agreements (e.g., UN; Council of Europe; American Conventions, etc.). Reported case law will not be reviewed because it is a large field that would warrant a separate project with a different approach.

##### 3.1.1.1 Review Objective 1: Mapping definitions of hate crime, hate incidents and hate speech

To achieve *objective 1*, we will include documents that (1) focus specifically on defining and conceptualizing “hate crime,” “hate speech,” “hate incidents” or any surrogate terms that are used to capture these concepts OR (2) focus specifically on hate crimes, hate incidents, hate speech or a surrogate for these terms AND (3) offer a specific definition of hate crime, hate speech, hate incidents or a surrogate for these terms.

How are definitions presented? We will recognize definitions as statements that describe what is meant by hate crime, hate incident, hate speech (and surrogate terms). Definitions can be found most likely in the introduction or methods section of a paper, in footnotes, or in a specific section of a gray literature report. Definitions are oftentimes more clearly delineated in the legal literature and statutes. The documents might explicitly state that they are “defining” the term (e.g., “we define hate crime as … “), or directly describe what is meant by the term (e.g., “hate crime is …”). Definitions of key multinational organizations (such as the United Nations (and specific committees such as the UN Committee on the Elimination of Racial Discrimination) and the Organization for Security and Co‐operation in Europe) will also be included in the review.

In relation to objective 1, this review will include:

Academic literature:

- Empirical articles, with any study design, which propose an original definition of hate crime, hate speech, hate incidents or surrogate terms. We include studies with all study designs because definitions and measurement tools can appear in documents presenting all types of empirical research.
- Theoretical articles that focus specifically on defining hate crime, hate speech, hate incidents or surrogate terms

Gray literature:

- Reports authored by government and nongovernment organizations, which propose an original definition of hate crime, hate speech, hate incidents or surrogate terms. We expect this literature to include policy and programming areas including political laws, civil acts and codes highlighting the criminality in discriminative actions such as hate speech and hate crime.
- Tech companies' definitions of hate speech and hateful conduct in terms of service, community standard guidelines and transparency reports. Specifically, we will include the members of the Global Internet Forum to Counter Terrorism (GIFCT), which includes 17 members as of May 2021.

Legislation:

- Current criminal, civil or human rights legislation that is intended to regulate or allow for the collection of data on forms of hate speech, hate incidents and hate crimes.

##### 3.1.1.2 Review Objective 2: Mapping tools to measure the prevalence of hate crime, hate incidents and hate speech

In relation to objective 2, we will include empirical articles, with any study design, which measure the prevalence of hate crime, hate speech, hate incidents or surrogate terms.

What does a measurement tool look like? A measurement tool is an operationalization of a definition of any of the concepts of hate crime, hate speech and hate incidents in the form of interview/survey/focus group questions, community reporting tools, coding schemes for visual/audio/textual content, among others.

In relation to objective 2, we will include studies that take an existing definition of hate and operationalize it in an original way (which means, if study X used Y's tool, we exclude X and only include Y), use samples related to one of the countries under investigation in this review (see Population section), and provide a transparent description of the parameters (measures) or indices used for measurement.

We will include studies that might assess reliability and/or validity of the original measurement tool. Empirical studies—aside from those focusing on the development of a tool—will be included. For example, there are many articles that have used Uniform Crime Reporting (UCR) statistics on hate crimes or National Crime Victimization Survey (NCVS) data, but often in very different ways: operationalizing variables in different ways. Gray documents such as reports from government agencies (e.g., Canada's Statistics Canada, Canadian Centre for Justice and Community Safety Statistics) will be included. A mere description of the tool is sufficient for inclusion, but measures of validity and reliability will be taken into consideration when present and it is one aspect we take into account in our review of measurement tools in accordance with Objective 2.

#### 3.1.2 Population

We focus on Canada and a sample of countries in different regions that are comparable in terms of democratic institutions, socio‐political context and legislative approach to hate crime, hate speech and hate incidents.

Eligible countries include, in addition to Canada, the United States and the United Kingdom, which represent two key and different approaches to hate crime data collection globally, with the United States generally requiring a crime to present objective indicators of bias to be counted as a hate crime, and the United Kingdom accepting third party perceptions as a valid hate crime indicator at the policing stage (not at the prosecutors or court stage). We include Ireland, Germany, France, Italy and Spain, which represent a heterogeneous sample of European countries in terms of hate crime and hate speech legislation, different socio‐political contexts and approaches to hate crime and hate speech data collection, as identified by recent reports by the European Union Agency for Fundamental Rights and the European Parliament. For example, Germany and Italy have unique hate speech regulations to target hate speech propaganda and hate crime associated with the Nazi and Fascist ideologies. Ireland and Spain include third party perceptions as bias indicator, while France and Germany do not, and Italy does not have a list of bias indicators. Germany and France require by law to record only specific forms of hate crime, Spain and Ireland incorporated hate crime in the general crime recording system, but in Spain recording is not compulsory while in Ireland it is, and in Italy hate crime is not incorporated in the general recording system although hate crime data is collected by law enforcement authorities. All five countries have hate crime and hate speech regulations in criminal law, civil law, media law and press‐self regulation, as well as watch‐group organizations collecting hate incidents reports, which make them relevant case studies. We include Australia, which is a comparable context with Canada in terms of legal, political and democratic structures (e.g., federalism, bicameral parliaments, common law legal system—although Canada has one criminal law, whereas in Australia there are different statutes in each state). Moreover, Canada and Australia have striking similarities in terms of geographical and demographic distribution (i.e., population concentration in urban areas), economic and social indicators (e.g., GDP per capita, unemployment), cultural and linguistic indicators, migrant population and attitudes to diversity, social and political issues (such as relationships between First Nations and European settlers), all of which constitute significant predictors of intergroup conflict that shape the types and forms of hate crime, hate incidents and hate speech taking place in Australia and Canada.

We include New Zealand, which after the Christchurch attack significantly increased its efforts in policy, practice and research on hate behaviors, as demonstrated by the Christchurch call to eliminate terrorist and violent extremist content online, and by the Royal Commission of Inquiry on the Christchurch attack, and its numerous recommendations relevant to regulating hate crime, hate speech and hate incidents.

The review will take into consideration academic articles in English, French, German, Italian and Spanish languages, and will review relevant contemporary legal statutes, policy and programming in North America (Canada and United States), Europe (Germany, France, United Kingdom, Ireland, Italy and Spain), Australia, and New Zealand. We focus on these languages because we aim to capture relevant documents in the countries that are included in the review.

#### 3.1.3 Phenomenon of interest

This review focuses specifically on capturing both general definitions of hate crime, hate speech and hate incidents; and definitions of surrogate terms capturing hate directed towards identities based on the following perceived characteristics that appear in Canada's legislation:

1. Racial and ethnic identity (e.g., racism, xenophobia, sinophobia);
2. Religious identity (e.g., anti‐Semitism, anti‐Muslim);
3. Sexual orientation (e.g., homophobia, lesbophobia, biphobia);
4. Gender identity (e.g., sexism, misogyny, transphobia);
5. Disability (e.g., ableism, disablist violence);
6. Occupation identity (e.g., anti‐abortion violence).

Our review will also pay particular attention to how protected characteristics are defined and captured, and how other characteristics absent from lists are captured by existing definitions and measurement tools. In this review, we focus exclusively on definitions of malicious behaviors motivated by (or demonstrating) prejudice, or by a worldview or ideology that dehumanizes the target group and justifies aggression and violence, and how these definitions are operationalized to measure these behaviors. Behaviors that will be included in this review can be criminal behaviors (e.g., murders, violence against people and properties, and forms of hate speech regulated by the criminal code) and noncriminal behaviors (e.g., the so‐called “protected hate speech” in the United States or other jurisdictions). Both on‐ and offline behaviors are included in our scope. Discrimination (e.g., discrimination in workplace, sale and supply of services), public order offences, sex crimes and harassment are excluded because they are different fields of literature, which we believe would need a separate independent review, especially in relation to the legal literature.

#### 3.1.4 Context

Our review focuses on definitions and measurement “hate crime,” “hate speech,” “hate incidents” or any surrogate terms that are used to capture these concepts. Much of the literature focusing on “hate” and surrogate terms (such as anti‐Semitism, religious hate, racial hate, etc.) uses these terms to define both behaviors and attitudes (e.g., negative attitudes to a racial or religious group). This review will only focus on definitions and measurement tools that focus on capturing behaviors including speech.

Some of the literature focusing on concepts like homophobia, Islamophobia or anti‐Semitism, focuses on negative attitudes to out‐groups instead of behaviors (see e.g., Huynh et al., 2020; Uenal et al., 2020). This area of literature focusing on attitudes, although using concepts that might be relevant for this review (such as homophobia, Islamophobia, and anti‐Semitism), will be excluded because our main focus are definitions and measurements of behaviors, not cognitive activities. As we only focus on measurement tools capturing behaviors only (not attitudes), we will not take into consideration any psychometric scale instrument, or any tool aiming to capture attitudes related to hate or surrogate terms (e.g., racism, homophobia, anti‐Semitism, etc.).

### 3.2 Search methods for identification of studies

#### 3.2.1 Electronic searches

We developed our search strategy based on Kugley et al. (2016) methods guide on search strategies, discussions within our team, with Campbell Collaboration, as well as with input from members of our External Advisory Board and university librarians. We will use the same search strategies to identify literature relevant to objective 1 and objective 2. However, our search strategies to identify different types of documents (i.e., academic and gray literature, federal and state statutes; see above) will be tailored to optimize the retrieval of potentially relevant documents for each type and, where practicable, each country. In selecting the databases for electronic searches, we aim to provide a broad coverage by including multi‐disciplinary databases and citation indexes as well as subject‐specific databases to specifically identify potentially relevant documents in French, German, Spanish and Italian (hereafter: databases). For the purpose of our review, we do not consider it relevant to include trials registers in our search strategy because our review is not a review of interventions. Please find a full list of databases in Table 2 and the specific search strategies in Supporting Information Annex 3.

For electronic searches, we have devised a search strategy that combines search terms grouped into three categories:

1. Attribute‐specific search terms: these search terms correspond to groups that commonly become the target of hate crimes, hate incidents or hate speech;
2. Behavior‐specific search terms: these search terms correspond to the term used to describe the behavior in question, such as crime, or language;
3. Country‐specific search terms.

These search terms capture our broad focus on hate crime, hate incident and hate speech as well as surrogate terms. We decided against search terms that would reflect our research objectives, such as “definition” or “measurement,” because we would expect that doing so may exclude potentially relevant documents from search results, similarly to Campbell et al. (2014) observations. As we do not have specific eligibility criteria around study design, we do not consider it necessary to include study design as a criterion in our search strategy. Please see Table 1.

**Table 1:** Search terms for electronic searches^a^

Attribute		Behavior	Geographical context
Generic	hate	crim*	“United States”
	prejudice*	speech	“US”
	bias*	incident*	“USA”
Racial and ethnic	racis*	conduct	Australia*
	xenophobi*	act	“New Zealand*”
	sinophobi*	abus*	Aotearoa
	anti‐foreigner	vilif*	France
	anti‐migrant*	language*	French
	anti‐immigrant*	violen*	German*
	anti‐refugee*	rape*	Irish
	“anti‐asylum seeker”	murder*	Ireland
	anti‐Roma	harass*	Ital*
	anti‐traveller	terroris*	Spain
	anti‐Gypsy	narrative*	Spanish
	“anti‐First Nations”	discourse*	“UK”
	anti‐Indigenous	propaganda	“United Kingdom”
	anti‐Maori	“targeted violence”	Brit*
	anti‐Aboriginal	incite*	Engl*
Religious	islamophobi*	extremis*	“Northern Ireland”
	antisemiti*	hostil*	“Northern Irish”
	anti‐Semiti*	micro‐aggression	Scot*
	anti‐Jew*	microaggression	Wales
	anti‐Amish	group*	Welsh
	anti‐Sikh		Canad*
	anti‐Buddhis*		
	anti‐Muslim*		
	anti‐Islam*		
	anti‐Christian*		
LGBTIQ + and non‐binary	homophobi*		
	transphobi*		
	lesbophobi*		
	biphobi*		
	anti‐gay		
	anti‐lesbian*		
	anti‐bisex*		
	anti‐transgender		
	anti‐LGBT*		
Disability	ableis*		
	disableis*		
Gender	sexis*		
	misogyn*		
	misandr*		
	gender‐based		
	“gendered”		
	incel		
	invcel		
	“involuntary celibate”		
	anti‐feminis*		
Occupation	anti‐abortion		
	anti‐doctor		
	“anti‐sex worker”		
	anti‐politician		

^a^ Please note that the terms are designed to capture how hate behaviors are defined in the existing literature, they do not reflect terminology choices by the research team. Searches will be performed by combining all “attributes” with all “behaviors” and all “geographical contexts.”

For each electronic search, we will adapt our search strategy depending on the capacities and specificities of the search function of the database in question. We divided the databases into two groups: the first group of databases allows us to combine all our search terms with Boolean and proximity operators into a single search string; the second group of databases has limited search functionalities which do not allow us to use our comprehensive search string. Please find a list of databases grouped into the two categories in Table 2.

**Table 2:** List of databases for electronic searches grouped by search functionality

Group 1: Databases with complex search capacities	Group 2: Databases with limited search capacities
Databases hosted by EBSCOhost	Science Direct
Communications and Mass Media Complete	Google Scholar
Criminal Justice Abstracts	National Criminal Justice Reference Centre Abstracts Database (https://www.ojp.gov/ncjrs/virtual-library/search)
SocIndex	Publications Office of the European Union (https://op.europa.eu/en/home)
Databases hosted by ProQuest	EUR‐Lex (https://eur-lex.europa.eu/homepage.html)
Continental Europe Database	Organisation for Security and Economic Co‐operation in Europe, Office for Democratic Institutions and Human Rights Document Library (https://www.osce.org/resources/documents)
Dissertation and Theses Global	United Nations Digital Library (https://digitallibrary.un.org/?ln=en)
ERIC	United Nations Digital Library (https://digitallibrary.un.org/?ln=en) United Nations Office of the High Commissioner for Human Rights Digital Library (https://searchlibrary.ohchr.org/)Torrossa
Sociological Abstracts	
Technology Collection	
Databases hosted by Web of Science	
Web of Science Core Collection	
SciELO Citation Index	
Databases hosted by Ovid	
PsycInfo	
Scopus	

Each search strategy for databases falling into group 1 includes the use of placeholders and wildcards as well as loose/approximate and exact phrases. Additionally, we will use search limits and filters, where possible, to filter out material published before 1990, as this material is beyond the scope of our review. We focus on materials published after 1990 because it is the point at which both policy responses and academic attention to hate motivated behaviors began in earnest. The Hate Crime Statistics Act (HCSA) of 1990 set into motion the structure and mechanisms for identifying and collecting hate crime data in the United States, and corresponded with a surge in academic publications on hate crime globally. For this reason, a number of hate crime data collection efforts, like Gruenewald's Bias Homicide Database (BHDB), begin in 1990.

The search terms within each column will be connected with “OR” operators; attribute‐specific and behavior‐specific search terms will be connected with a proximity operator where available, such as “W/n” in Scopus, or otherwise with an “AND” operator; and behavior‐specific and country‐specific search terms will be connected with an “AND” operator. We have chosen to use a proximity operator, where available, to connect attribute‐ and behavior‐specific search terms to improve the precision of our searches.

For databases falling into group 2, our search strategy will be limited to eight keywords:

1. “hate crime”
2. “hate speech”
3. “hate incident”
4. “hate conduct”
5. “hate propaganda”
6. “hate group”
7. “prejudice‐motivated crime”
8. “bias crime”

We chose these keywords because they are the most commonly used in the literature published in English, and because we expect them to have equivalents in the other languages considered in our review in the form of direct translations. For searches carried out in Italian, Spanish, French and German, we will translate those keywords into the respective languages. Where possible, we will combine these search terms in a single search combining them with the Boolean operator OR. Where this is not possible, we will carry out individual searches for each term separately. Please find the individual search strategies for each database in groups 1 and 2 in Supporting Information Annex 3. For the purpose of our review, we do not consider the use of search filters, such as the Cochrane Highly Sensitive Search Strategies for identifying randomized trials in MEDLINE, appropriate.

We will conduct one round of forward citation searching in Google Scholar of the academic studies that will be included in the review.

For each electronic search, we will document the following details to ensure reproducibility of our search strategy:

- Date of the search (dd/mm/yyyy)
- Name of the database
- Search string used (copied and pasted from the search field)
- Number of search results

#### 3.2.2 Searching other resources

To complement electronic searches, we will use complementary search strategies to minimize the risk of bias. These search strategies include:

(1) Hand‐searching of reference lists of any systematic reviews on hate crimes, hate incidents, hate speech, or a surrogate of these terms identified throughout the review process.
(2) Hand‐searching of reference lists of any documents included in the review, including systematic reviews.
(3) Liaising with our External Advisory Board to identify stakeholders in relevant fields with the aim of identifying potentially relevant documents, including unpublished or ongoing projects, key government and nongovernment organizations producing relevant gray literature, and legislation/statutes in each country context.
(4) Search websites of relevant organizations and stakeholders for potentially relevant documents. We aim to identify relevant organizations and websites based on our previous research and existing professional networks, through hand‐searching of included documents, and based on feedback from our External Advisory Board. Please find a list of websites that we will search in Supporting Information Annex 4. Organisations with document libraries that allow for basic searches are included in Table 2.

To ensure reproducibility of our search strategy, for each search carried out through means other than electronic searches, we will record:

- Date the search (dd/mm/yyyy)
- Means (e.g., through hand‐search of reference list of another document, through referral from External Advisory Board) and full details of search location
- Name of researcher who carried out the search
- Total number of search results

To identify current federal and state statutes, we will draw on key gray literature providing the full list of current legislation regulating hate crime, hate speech and hate incidents in a given country. An example is the report of the Royal Commission of Inquiry into the Terrorist Attack on Christchurch Mosques on March 15, 2019, which identifies and lists all “Hate speech and hate crime related legislation.” For each country, we will develop a list with relevant current legislation based on this strategy. Prior to coding, we will send these lists to academic experts of hate crime and hate speech legislation and legislation practitioners' from each country of focus for review.

### 3.3 Data collection and analysis

#### 3.3.1 Description of methods used in primary research

##### Review Objective 1: Definitions

What follows are exemplars of the types of papers to be included in the review and their uses.

In Fuentes Osorio (2017), the author examines the factors that have given rise to and shaped the development of the concept of hate crime and their ongoing implications for legal frameworks governing hate crime based on an analysis of relevant legislation and court cases. In his work, he draws on the following definition of hate crime:

Los “delitos de odio” se refieren inicialmente a delitos clásicos agravados por la motivación del sujeto activo y/o por la selección discriminatoria del sujeto pasivo. [“Hate crimes” initially refer to classic crimes that are aggravated by the motivation of the perpetrator and/or the discriminatory selection of the victim.] (Fuentes Osorio, 2017, p. 2).

This definition of “hate crime” outlines three key criteria: first, hate crime is a “classic crime,” that is, the behavior in question is defined as criminal under the law; second, hate crime is identifiable by the perpetrator's motivation to engage in criminal behavior; third, hate crime is identifiable based on the discriminatory nature of the choice of the victim. This study meets our inclusion criteria (2) and (3) in relation to objective 1, as it focuses specifically on hate crimes, hate incidents, hate speech or a surrogate for these terms and offers a specific definition of hate crime, hate speech, hate incidents or a surrogate for these terms.

##### Review Objective 2: Measurement tools

In Benier et al. (2016), the authors explore the applicability of ecological theories of hate crime developed in the United States for the Australian context. The authors draw on established definitions of hate crimes by Green et al. (2001), Victorian Equal Opportunity and Human Rights Commission (2010) and the FBI (2013) as

[…] unlawful, violent, destructive or threatening behavior in which the perpetrator is motivated in whole or in part by prejudice towards the victim's per‐ceived race, ethnicity, religion, sexual orientation, gender identity, age, impairment or homelessness (Benier et al., 2016, p. 479).

The authors operationalize this definition in the form of survey questions on direct and indirect victimization experiences for a range of crimes, including break‐and‐enter of private residences, various forms of property damage as well as muggings, assaults and sexual assaults. The authors provide the following as an example of such a survey question (Benier et al., 2016, p. 485):

While you have lived in this community, has anyone ever used violence such as in a mugging, fight or sexual assault against you or any member of your household anywhere in the community?

Survey respondents who answered this question with “yes” were then asked whether they believed the incident had occurred because of the victim's skin color, ethnicity, race or religion; only participants who answered both questions with “yes” were classified as reporting hate crime victimization, regardless of whether they reported direct or indirect victimization (Benier et al., 2016, pp. 485–486). Using multi‐level logistic regression models, the authors estimated the effects of household‐ and neighborhood‐level variables on the likelihood of hate crime victimization in Brisbane neighborhoods. This study meets our inclusion criteria (1), (2), and (3) in relation to objective 2 because it presents empirical research, identifies how hate crime, hate speech, hate incidents or a surrogate for these terms are operationalized and describes the type of data employed or collected to study these terms and the method used to analyze the data.

### 3.4 Selection of studies

Within our research team, we have divided responsibilities for the title/abstract as well as full‐text screening and coding of potentially relevant documents based on expertise, anticipated volume of potentially relevant documents and language knowledge. The groups are as follows: Germany and USA; Canada, France, Great Britain and Ireland; Spain, Italy, Australia, and New Zealand. This is relevant insofar as it relates to the way we map out our screening and coding processes, as detailed below.

We will use EndNote and Zotero to manage all documents retrieved throughout the search process. The use of Zotero, however, will be limited to researchers who may not have access to EndNote, for the manual entry of legislation and gray literature documents. All academic documents will be imported into Endnote.

Legislation will undergo an expert review instead of title/abstract screening process. Upon completion of the search process, each country team will compile a list of legislation that it has retrieved and send this list to a legal scholar or practitioner with expertise in the respective country for review. We will provide the expert with a summary of our project and its objectives and ask them to remove any legislation from our list that is outdated and replace it with most up‐to‐date legislation. Subsequently, we will import all legislation into EPPI Reviewer for full‐text screening.

All academic and gray literature citations will be imported from Endnote and Zotero into EPPI Reviewer to conduct title/abstract and full‐text screening. Prior to screening, all duplicates will be removed in EPPI Reviewer.

We will use EPPI Reviewer's machine learning functionality “Priority Screening” to support title/abstract screening. Because we include documents in English, French, German, Italian and Spanish, we will progress through title/abstract screening in stages. In a first step, we will import all citations in English into EPPI Reviewer and remove all duplicates. In a second step, we will set up “Priority Screening” in EPPI Reviewer and “train” the machine learning algorithm based on a random sample of citations. To do so, each title/abstract in the random sample will be screened independently by the whole team researchers, with any disagreements resolved during team meetings. As we seek to combine the “training” of the machine learning algorithm with achieving intercoder reliability of 90% or higher. Once we have achieved the aforementioned level of intercoder reliability and the algorithm is considered to be working effectively, we will proceed with title/abstract screening of all citations in English. To complete the title/abstract screening for citations in English, each citation will be screened by one researcher assisted by “Priority Screening.”

Upon completing title/abstract screening for citations in English, we will conduct the title/abstract screening for citations in French, German, Italian and Spanish in a second stage. Our team has at least one speaker for each of the languages other than English. One person will train EPPI Reviewer's ML algorithm and then use the “Priority Screening” in these languages when the minimum requirements for ML training are met. Effectively, the ML functionality will act as a second screener for the corpus in languages other than English. To check for accuracy, when Title & Abstract screening will be completed, we will undergo a random checking of 5% of the documents in languages other than English.

#### 3.4.1 Review Objective 1: Definitions

Inclusion and exclusion criteria for objectives 1 and 2 will be identical for title/abstract screening. Specific additional criteria will be added for full‐text screening. The inclusion criteria for title/abstract screening are provided in Table 3.

**Table 3:** Inclusion criteria for title/abstract screening

*Inclusion criteria*
Document was published in or after 1990
Document explicitly focuses on hate crime, hate incidents and/or hate speech or a surrogate for these terms
Document focuses on hate crime, hate incidents and/or hate speech or a surrogate for these terms in a geographical context within the scope of this review (Canada, USA, UK, Ireland, France, Germany, Spain, Italy, Australia and New Zealand)
Document focuses on behaviors that can be classified as hate crime, hate incidents and/or hate speech (examples are: murder, physical aggressions, property damage, vandalism, graffiti, offensive gestures, offensive or abusive social media posts).
Document can be classified as academic literature, gray literature, legal literature or federal and state statutes.

Documents to be included in our review in relation to objective 1 need to meet the following inclusion criteria for full‐text screening (Table 4).

**Table 4:** Inclusion criteria for full‐text screening of documents containing definitions of hate crime, hate incidents and hate speech or surrogate terms

*Inclusion criteria*
Document was published in or after 1990
Document can be classified as academic literature, gray literature, legal literature or federal and state statutes.
Document focuses on hate crime, hate incidents and/or hate speech or a surrogate for these terms in a geographical context within the scope of this review (Canada, USA, UK, Ireland, France, Germany, Spain, Italy, Australia and New Zealand)
Document includes an original definition, that is, a definition that is not an identical copy of existing definitions.

To avoid confusion about whether any document is included or excluded in relation to objective 1, coders will be asked to assign a code upon making their decision that will label whether a document was included or excluded in relation to objective 1, objective 2, or both.

#### 3.4.2 Review Objective 2: Measurement tools

The procedure for title/abstract screening for documents in relation to objective 2 will be identical to that outlined above for documents in relation to objective 1. However, consistent with the inclusion criteria outlined above, the inclusion criteria for full‐text screening will differ. These criteria are provided in Table 5.

**Table 5:** Inclusion criteria for full‐text screening of documents containing measurement tools of hate crime, hate incidents and hate speech or surrogate terms

*Inclusion criteria*
Document was published in or after 1990
Document can be classified as academic literature, gray literature, legal literature or federal and state statutes.
Document describes how hate crime, hate speech, hate incidents or a surrogate are measured (i.e., measurement parameters are transparent).
Document focuses on hate crime, hate incidents and/or hate speech or a surrogate for these terms in a geographical context within the scope of this review (Canada, USA, UK, Ireland, France, Germany, Spain, Italy, Australia and New Zealand)
Document includes an original measurement tool, that is, a measurement tool that is not an identical copy of existing measurement tools.

To avoid confusion about whether any document is included or excluded in relation to Aim objective, coders will be asked to assign a code upon making their decision that will label whether a document was included or excluded in relation to objective 1, objective 2, or both.

Our team will include documents in English, French, Italian, Spanish, and German. Because these are the languages spoken in the countries that we focus on, and will therefore capture virtually all relevant documents. Documents in other languages will be excluded.

### 3.5 Data extraction and management

The full coding form is attached in Supporting Information Annex 2. For all documents, we will extract information about authors, year, document search location, document type (academic—and if so, the discipline—legal and policy/gray), country of primary focus of the document (which can be multiple if a comparative document), language, key focus (whether hate crime, hate incident, hate speech or surrogate term), whether the article describes findings from empirical research, and whether it contains definitions or measurement tools. We will provide training to all coders and before starting the full coding we will test intercoder reliability on a selected sample to ensure the reliability of the coding. As with title/abstract and full‐text screening, we will establish intercoder reliability based on a random sample of documents in English.

Each document in the random sample will be coded independently by all researchers who participate in data extraction; disagreements will be resolved in team discussions. Further coding to achieve intercoder reliability of 90% or higher will be conducted as required and until at least 90% intercoder reliability are achieved.

#### 3.5.1 Review Objective 1: Definitions

To achieve objective 1, for each document we will extract information about the components of the definition of hate crime, hate speech, hate incidents and surrogate terms. *Components* include: (1) how the motivation is named (e.g., racist, Anti‐Semitic, hate, etc.); (2) how the nature of the behavior is named (e.g., crime, incident, speech, act, etc.); (3) how the target is described (e.g., a person, a property, a person and property, etc.); (4) whether perception is a bias indicator; and (5) the protected characteristics (if any). Additionally, we will code *categorical variables identifying whether*:

- the definition is adopted by any governmental or nongovernmental organization or linked to any statute or legislation;
- the definition allows for capturing different degrees of bias motivation;
- the victim of the hate behavior is assumed to be interchangeable;
- the definition reflects one of the two models described by Lawrence (1999) “discriminatory selection model” or “animus model.”

#### 3.5.2 Review Objective 2: Measurement tools

To achieve objective 2, for each document we will extract information about: the type of measurement *tool*, defined as a vehicle or an aid to collect information and data (e.g., an online module to collect data about hate incidents, or an automated text analysis algorithm, or a survey); the *metric* s used to measure hate, defined as parameters (measures) or indices used for measurement, comparison or tracking performance (e.g., bias indicators); and the *methods* used to measure hate, defined as the process and approach involved in a systematic inquiry of hate crimes, hate incidents, hate speech or surrogate terms, and generally refer to study design or application of an analytical method to this topic.

We will code categorical variables looking at information about the target identities that the measurement tool encompasses, the bias indicators used (if any), whether it is adopted by any government or nongovernment organizations, the variables collected about the incident, the victims, and the offender, and the indicators used to assess the severity of the crime. If there is *any analysis of quantitative data collected using the measurement tool*, we will collect—when present—information about context and sample, data collection methods, data analysis, and any reporting about feasibility, efficacy, reliability or validity of the instrument. We will also collect information about whether the instrument is at concept development, pilot or implementation stage.

Based on the OMERACT tool to assess quality of measurement tools (Beaton et al., 2019), we will collect information relative to the domains of truth (i.e., does the instrument cover all the protected characteristics present in the relevant legislation), feasibility (i.e., is the instrument accessible? Is it available in multiple languages?) and discrimination (is there stability in situations of no change? Does the instrument detect score change in situation of real change? How well does the instrument distinguish between groups?). We expect that, while information about truth and feasibility might be available for most instruments, information about discrimination might not.

### 3.6 Assessing the methodological limitations of included studies

We propose the following criteria that draw mostly on assessments of theories and concepts (for objective 1) and gap maps of tools, metrics and methods (for objective 2).

#### 3.6.1 Review Objective 1: Definitions

To assess the quality of definitions, we will conduct post‐hoc analyses of the definitions against the following criteria:

1) Does the definition contain unambiguous concepts (Ritzer, 1991)? For example, we will look at whether the hate motivation is clearly defined (e.g., is the threshold between hateful and non‐hateful behavior clearly defined?) and at whether there is a clear understanding of how to identify the hate motivation (e.g., what are the bias indicators?)
2) Is there a justification or rationale for inclusion of a certain element or component of the definition? For example, some definitions have protected characteristics, and others do not. Is there a clear rationale for having vs not having protected characteristics?
3) Is the definition adopted by governmental or nongovernmental organizations, or current statutes? We will look for this information inside the document.

We will operationalize these criteria as three separate dimensions to assess the quality of definitions by attributing a score from 1 (strongly disagree), 2 (disagree), 3 (agree), 4 (strongly agree) to each.

#### 3.6.2 Review Objective 2: Measurement tools

To assess the quality of measurement tools, we use the approach described by Sparling et al. (2019) in their gap map of tools, metrics and methods in the field of food systems and agriculture‐nutrition. First, we will look at whether instruments are at different stages of development, which include: (1) concept development; (2) pilot; and (3) wide‐spread application. Second, we will look at whether the documents report any information about (1) feasibility, efficacy or internal validity of the instrument, and (2) validity or reliability of the instrument. Third, we will consider how much information about the hate incident is included in the instrument (e.g., about the offender, victim, incident, bystanders, etc.) and whether the instrument is adopted by any governmental or nongovernmental organization. Finally, we will collect information relative to the domains of truth, feasibility and discrimination (Beaton et al., 2019). Where present, we will report reliability and validity of measurement tools included in this review, using the template in Wells et al. (2009).

### 3.7 Description of data to be mapped

#### 3.7.1 Review Objective 1: Definitions

Given the qualitative textual nature of the information extracted (see Data extraction and management section) we will attribute categorical codes to the text extracted. The unit of analysis will be the definitions. For each definition, we will code components (e.g., attribute, behavior, protected characteristics, etc.) and other additional qualitative categories (e.g., whether the definition is adopted by any government or nongovernment organization, whether it allows for degrees of motivation, etc.).

Specifically, to answer each research question, the following information will be examined:

○ How are hate crimes, hate speech and hate incidents defined in the academic, legal, policy, and programming literature? We will look at definition components and other qualitative categories, and document type (whether academic, gray or legal, and—if academic—which discipline it is situated in).
○ What are the concepts, parameters and criteria that qualify a behavior as being hate crime, hate incident or hate speech? We will look at definition components and other qualitative categories—such as whether a definition defines a behavior as a hate crime by establishing its motivation or by looking at whether the behavior displays a hate element.
○ What are the most common concepts, parameters and criteria found across definitions? What are the differences between definitions and the elements they contain? We will look at definition components and other qualitative categories, and country, language, document type, year, and affiliation of the author.

#### 3.7.2 Review Objective 2: Measurement tools

The majority of the information that we will collect about the measurement tools will be qualitative and textual (see Data extraction and management section), and we will attribute categorical codes to the text extracted. For each instrument, we will code tools, metrics and methods, stage of development and additional qualitative information (e.g., whether the instrument is adopted by any government or nongovernment organization).

Additionally, when reported, we will extract additional information about sample, context, and any available information about feasibility, efficacy, reliability and validity of the instrument. Specifically, to answer each research question of the review we will look at how are definitions operationalized to measure hate crimes, hate speech, and hate incidents? We will code tools, metrics and methods. We will also look at how valid and reliable are these measures? We will look at stage of development and additional qualitative information, as well as any available information about feasibility, efficacy, reliability and validity of the instrument, when reported.

### 3.8 Data mapping

Final tables and figures will be decided based on the findings. The report will include a section discussing under‐researched areas and gaps. Tables and figures will include a PRISMA diagram, and might include a visual representation of trends in definitions and measurement tools, and a visual and numeric summary of definitions and measurement tools highlighting the most and least common components and instruments. The report will include a narrative section where we will interpret and contextualize the existing literature within paradigms and historical and political contexts to develop new understandings of definitions and measurement tools. If possible, we will aim to develop typologies of definitions and measurement tools.

#### 3.8.1 Review Objective 1: Definitions

The unit of analysis will be the definitions. We plan to present univariate analysis of the categorical data, as well as bivariate analysis (e.g., cross‐tabulations, bar charts) of definition components and additional qualitative categories with document type, academic field, country, language, year, and authors' affiliation (whether a university, a government or nongovernment organization). We will also present a narrative report describing definition components and additional qualitative categories, which will be used to provide an assessment of the definitions (see section Assessing the methodological limitations of included studies).

The full list of the analyses that will be performed is available in Supporting Information Annex 1.

#### 3.8.2 Review Objective 2: Measurement tools

The unit of analysis will be the instruments to measure hate speech, hate incidents, hate crimes or surrogate terms. We plan to present univariate analysis of the data that will be collected and coded as part of this review, which includes categorical data, as well as bivariate analysis (e.g., cross‐tabulations, bar charts) of instruments, metrics, tools, methods and additional qualitative categories with document type, academic field, country, language, year, and authors' affiliation (whether a university, a government or nongovernment organization). We will also present a narrative report describing instrument's characteristics, stage and additional qualitative categories, which will be used to provide an assessment of the definitions, together with any available information about the instrument's feasibility, efficacy, reliability and validity (see section Assessing the methodological limitations of included studies).

The full list of the analyses that will be performed is available in Supporting Information Annex 1.

### 3.9 Assessment and investigation of heterogeneity

This study will map the heterogeneity of the field under investigation, highlighting trends and differences between definitions and instruments adopted across geographical contexts, research and policy domains. This will be done by displaying visually, using different types of charts (e.g., heat maps, bar charts, line charts, see Sparling et al., 2019), the different types of definitions and measurement tools components that will be coded as described in the “Data extraction and management” section.

#### 3.9.1 Review author reflexivity

The team has a variety of disciplinary backgrounds that reflect different positions and approaches in the field of hate crime studies, including qualitative and theoretical sociology (Perry), quantitative criminology (Chermak and Freilich), mixed methods political psychology (Vergani) and mixed methods online research (Scrivens). The team is well versed in relevant theory and in the study of subfields relevant to this review, such as Islamophobia, anti‐Semitism, terrorism, and violent extremism. This review's aim is to map—not summarize or meta‐analyze—the existing definitions and measurement tools across different disciplines. The review team has been maintaining a reflexive position throughout all the stages of the review process, and decisions have been discussed critically and regularly among the team members with regular debriefing sessions to support with decision‐making and coding. Based on the team's collective and individual experiences, it is anticipated that the findings will reveal a combination of approaches and disciplinary contributions across the whole spectrum of social and psychological sciences relevant to the study of hate behaviors. The team will remain mindful of conscious and unconscious presuppositions and support each other to minimize the risk of these skewing our analysis or the interpretation of our findings. The research team is composed of researchers who greatly contributed to the literature under investigation in this review. For this reason, we will make sure that the authors of a document will not review and assess it.

## CONTRIBUTIONS OF AUTHORS

- Content: Vergani, Perry, Freilich, Chermak, Scrivens, Link
- Systematic review methods: Vergani, Perry, Freilich, Chermak, Scrivens, Link
- Information retrieval: authors and research assistants

## DECLARATIONS OF INTEREST

None.

## PRELIMINARY TIMEFRAME

The final report is due December 9, 2022.

## PLANS FOR UPDATING THE REVIEW

None.

## SOURCES OF SUPPORT

This review is funded by a Campbell Collaboration grant awarded to Vergani, Perry, Freilich, Chermak, Scrivens via Public Safety Canada.

## Supporting information

Supporting information.

Supporting information.

Supporting information.

Supporting information.
