# Supplementary material for: PROTOCOL: Mapping the scientific knowledge and approaches to defining and measuring hate crime, hate speech, and hate incidents
Source: Campbell Syst Rev. 2022 Apr 18;18(2):e1228. doi: 10.1002/cl2.1228 (PMC9014694; doi:10.1002/cl2.1228)
Supplement: Supplementary file 1 — Supporting information. [file CL2-18-e1228-s004.docx]

ANNEX 1.

Review Objective 1: Definitions

We will produce univariate analyses of all the variables. Data permitting, the following bivariate analyses will be performed:

- Document type (i.e. academic vs legal vs grey literature) by definition components (i.e. how the motivation is named, how the nature of the behavior is named, how the target is described, whether perception is a bias indicator, protected characteristics);
- Document type (i.e. academic vs legal vs grey literature) by definition attributes (i.e. whether the definition is adopted by any organisation or by legislation, whether the definition captures degrees of bias motivation, whether the victim is assumed to be interchangeable, whether it uses an animus or discriminatory selection model);Country by definition components (i.e. how the motivation is named, how the nature of the behavior is named, how the target is described, whether perception is a bias indicator, protected characteristics);
- Country by definition attributes (i.e. whether the definition is adopted by any organisation or by legislation, whether the definition captures degrees of bias motivation, whether the victim is assumed to be interchangeable, whether it uses an animus or discriminatory selection model);
- Document’s focus (i.e. hate speech vs hate incident vs hate crime vs surrogate term) by definition components (i.e. how the motivation is named, how the nature of the behavior is named, how the target is described, whether perception is a bias indicator, protected characteristics);
- Document’s focus (i.e. hate speech vs hate incident vs hate crime vs surrogate term) (i.e. whether the definition is adopted by any organisation or by legislation, whether the definition captures degrees of bias motivation, whether the victim is assumed to be interchangeable, whether it uses an animus or discriminatory selection model);

Univariate analyses will be presented in tables and using data visualisation (e.g. pie-charts, bar charts). Bivariate analyses will be presented by using cross-tabs and data visualization (e.g. bar charts, line charts). The best and most effective visualization will be decided by the research team when compiling the final report.

Review Objective 2: Measurement tools

We will produce univariate analyses of all the variables. Data permitting, the following bivariate analyses will be performed:

- Document type (i.e. academic vs legal vs grey literature) by the type of measurement tool (i.e. a vehicle or an aid to collect information and data, the metrics used to measure hate, and the methods used to measure hate);
- Document type (i.e. academic vs legal vs grey literature) by measurement tool components (i.e. the target identities that the measurement tool encompasses, the bias indicators used, whether it is adopted by any government or non-government organizations, the variables collected about the incident, the victims, and the offender, and the indicators used to assess the severity of the crime, and – if any analysis of quantitative data collected using the measurement tool is reported - context and sample, data collection methods, data analysis, and any reporting about feasibility, efficacy, reliability or validity of the instrument);
- Document type (i.e. academic vs legal vs grey literature) by domains of truth (i.e. whether the instrument covers all the protected characteristics present in the relevant legislation), feasibility (whether the instrument accessible and available in multiple languages) and discrimination (whether there is stability in situations of no change and how well does the instrument distinguish between groups);
- Country by the type of measurement tool (i.e. a vehicle or an aid to collect information and data, the metrics used to measure hate, and the methods used to measure hate);
- Country by measurement tool components (i.e. the target identities that the measurement tool encompasses, the bias indicators used, whether it is adopted by any government or non-government organizations, the variables collected about the incident, the victims, and the offender, and the indicators used to assess the severity of the crime, and – if any analysis of quantitative data collected using the measurement tool is reported - context and sample, data collection methods, data analysis, and any reporting about feasibility, efficacy, reliability or validity of the instrument)
- Country by domains of truth (i.e. whether the instrument covers all the protected characteristics present in the relevant legislation), feasibility (whether the instrument accessible and available in multiple languages) and discrimination (whether there is stability in situations of no change and how well does the instrument distinguish between groups);
- Document’s focus (i.e. hate speech vs hate incident vs hate crime vs surrogate term) by the type of measurement tool (i.e. a vehicle or an aid to collect information and data, the metrics used to measure hate, and the methods used to measure hate);
- Document’s focus (i.e. hate speech vs hate incident vs hate crime vs surrogate term) by measurement tool components (i.e. the target identities that the measurement tool encompasses, the bias indicators used, whether it is adopted by any government or non-government organizations, the variables collected about the incident, the victims, and the offender, and the indicators used to assess the severity of the crime, and – if any analysis of quantitative data collected using the measurement tool is reported - context and sample, data collection methods, data analysis, and any reporting about feasibility, efficacy, reliability or validity of the instrument);
- Document’s focus (i.e. hate speech vs hate incident vs hate crime vs surrogate term) by domains of truth (i.e. whether the instrument covers all the protected characteristics present in the relevant legislation), feasibility (whether the instrument accessible and available in multiple languages) and discrimination (whether there is stability in situations of no change and how well does the instrument distinguish between groups).

Univariate analyses will be presented in tables and using data visualisation (e.g. pie-charts, bar charts). Bivariate analyses will be presented by using cross-tabs and data visualization (e.g. bar charts, line charts). The best and most effective visualization will be decided by the research team when compiling the final report.
