# Supplementary material for: PROTOCOL: Mapping the scientific knowledge and approaches to defining and measuring hate crime, hate speech, and hate incidents
Source: Campbell Syst Rev. 2022 Apr 18;18(2):e1228. doi: 10.1002/cl2.1228 (PMC9014694; doi:10.1002/cl2.1228)
Supplement: Supplementary file 2 — Supporting information. [file CL2-18-e1228-s003.docx]

Coding tool

Start of Block: Metadata

Coder name

________________________________________________________________

Document complete citation

________________________________________________________________

Document weblink

________________________________________________________________

End of Block: Metadata

Start of Block: Article information

Document type

- Academic (please specify main discipline) (1) ________________________________________________
- Legislation (2)
- Grey literature (3)

Country (primary focus of the article)

- Canada (1)
- USA (2)
- Germany (3)
- France (4)
- UK (5)
- Ireland (6)
- Italy (7)
- Spain (8)
- Australia (9)
- New Zealand (11)
- Comparative (please indicate the focus-countries or region) (10) ________________________________________________

Language of the document

- English (1)
- German (2)
- French (3)
- Italian (4)
- Spanish (5)

| Page Break |  |
| --- | --- |

Key focus of the article (please note that some forms of hate speech, such as intimidation and threats, might be regulated by the criminal code and therefore be considered as hate crime, too). Please tick all that apply.

- Hate speech (1)
- Hate incident (2)
- Hate crime (3)
- Surrogate term(s) (4)

Display This Question:

If Key focus of the article (please note that some forms of hate speech, such as intimidation and th... = Surrogate term(s)

Please specify which surrogate term(s) are the key focus of the article.

________________________________________________________________

________________________________________________________________

________________________________________________________________

________________________________________________________________

________________________________________________________________

Does the article include a definition of hate crime, hate speech, hate incidents or a surrogate term?

- Yes (1)
- No (2)

Display This Question:

If Does the article include a definition of hate crime, hate speech, hate incidents or a surrogate t... = Yes

| 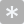 |
| --- |

If so, how many definitions of hate crime, hate speech, hate incidents or a surrogate term does the article include (min. 1, max. 5)?

________________________________________________________________

Display This Question:

If Document type = Academic (please specify main discipline)

How many citations does the document have on Google Scholar?

________________________________________________________________

Does the article include empirical research?

- Yes (1)
- No (2)

Does the article include a measurement of hate crime, hate speech, hate incidents or a surrogate term?

- Yes (1)
- No (2)

Display This Question:

If Does the article include a measurement of hate crime, hate speech, hate incidents or a surrogate... = Yes

| 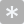 |
| --- |

If so, how many measurements of hate crime, hate speech, hate incidents or a surrogate term does the article include (min. 1, max. 5)?

________________________________________________________________

| Page Break |  |
| --- | --- |

End of Block: Article information

Start of Block: Explainer Definitions Loop & Merge

Display This Question:

If Does the article include a definition of hate crime, hate speech, hate incidents or a surrogate t... = Yes

You have indicated that the article contains ${Q2.7/ChoiceTextEntryValue} definition(s). We ask you to code each definition separately. You will be shown the same questions for each definition. Please code each definition individually and make sure that you only code information about the definition in question.

| Page Break |  |
| --- | --- |

End of Block: Explainer Definitions Loop & Merge

Start of Block: Definitions

**You are coding definition #${lm://CurrentLoopNumber}.**

| Page Break |  |
| --- | --- |

Please specify the author / proponent of definition #${lm://CurrentLoopNumber}'s NAME and AFFILIATION.

________________________________________________________________

Please copy and paste definition #${lm://CurrentLoopNumber} from document. If there is more than one definition in the document, please copy and paste (and code) one definition at a time.

________________________________________________________________

| Page Break |  |
| --- | --- |

Let's now look at the components of the definition.

How is the motivation named?

- Hate (4)
- Bias (8)
- Racist (1)
- Antisemitic (2)
- Islamophobic (3)
- Other (please specify) (5) ________________________________________________

How is the behaviour named?

- crime (1)
- incident (2)
- speech (3)
- conduct (4)
- violence (5)
- Other (please specify) (6) ________________________________________________

If the definition provides a list of behaviours that are considered (e.g. murder, vandalism, etc.) please tick all that apply or add in the "other" box the behaviours that are considered.

- The document does not provide a list of behaviours considered by the definitionfo (1)
- murder (2)
- nonnegligent manslaughter (3)
- forcible rape (4)
- robbery (5)
- aggravated assault (6)
- burglary (7)
- theft (8)
- arson (9)
- assault (10)
- intimidation (11)
- destruction / damage / vandalism of property (12)
- other (please specify) (13) ________________________________________________

What are the target identities that definition #${lm://CurrentLoopNumber} encompasses? Tick all that apply

- Generic (there is no specific identity) (1)
- Racial (please specify if any race is provided) (7) ________________________________________________
- Ethnic (please specify if any ethnicity is provided) (18) ________________________________________________
- National origins (please specify if any nationality is provided) (19) ________________________________________________
- Religious (please specify if any religion is provided) (8) ________________________________________________
- Sexual orientation (please specify if any sexual orientation is provided) (9) ________________________________________________
- People living with a disability (please specify if any disability is provided) (10) ________________________________________________
- Gender (please specify if any gender - including transgender - is provided) (11) ________________________________________________
- Sex (please specify if any sex is provided) (17) ________________________________________________
- Occupation (please specify if any occupation is provided) (12) ________________________________________________
- Age (14)
- Class (15)
- Housing status (16)
- Other (please specify) (13) ________________________________________________

How is the target of the behavior described?

- Person (1)
- Property (2)
- Organisation (3)
- Not named (4)
- Other (please specify) (5) ________________________________________________

Is the victim of the hate behavior assumed as being interchangeable?

- No (1)
- Yes (please copy and paste relevant section) (2) ________________________________________________
- Unsure (please specify why unsure) (3) ________________________________________________

| Page Break |  |
| --- | --- |

Let's now look at how the definition conceptualises the hate motivation.

Is the perception of the victim or a witness considered as a bias indicator in definition #${lm://CurrentLoopNumber}?

- Yes (1)
- No (2)
- Unsure (please specify why unsure) (4) ________________________________________________

Are there bias indicators associated with definition #${lm://CurrentLoopNumber}?

- Yes (1)
- No (2)
- Unsure (please specify why unsure) (4) ________________________________________________

Display This Question:

If Are there bias indicators associated with definition #${lm://CurrentLoopNumber}? = Yes

What are the bias indicators associated with definition #${lm://CurrentLoopNumber}? Please copy and paste a list if present

________________________________________________________________

Does definition #${lm://CurrentLoopNumber} allow for different degrees of bias motivation? For example, does the definition specify that a behavior can be motivated "in whole or in part" by hate?

- Not specified (1)
- No (4)
- Yes (please copy and paste the relevant section) in the box (2) ________________________________________________

Lawrence (1999) describes two models identifying the hate motivation: 1) a "discriminatory selection model", where it is required that the offender intentionally selected his or her victim from the protected group, and 2) a "animus model", which requires that the offender act out of hostility for the protected group. Which of the two models is reflected in definition #${lm://CurrentLoopNumber}, and why?

- Discriminatory selection model (please specify why) (1) ________________________________________________
- Animus model (please specify why) (2) ________________________________________________
- Unsure (please specify why unsure) (3) ________________________________________________

| Page Break |  |
| --- | --- |

Is definition #${lm://CurrentLoopNumber} proposed in the document based on previous definitions? If so, please add the references as they appear in the document. 
Please include references only if they appear in the same paragraph where definition # ${lm://CurrentLoopNumber} is provided.

________________________________________________________________

| Page Break |  |
| --- | --- |

Let's now look at the definition uptake.

Is definition #${lm://CurrentLoopNumber} adopted by any governmental or non governmental organisation?

- Not adopted by any organization (9)
- Not specified (8)
- Law enforcement (1)
- Human rights organisation (2)
- Community organisations (3)
- Private company (4)
- Official government survey (e.g. victimization survey) (5)
- Legislation (6)
- Other (please specify) (7) ________________________________________________

Is definition #${lm://CurrentLoopNumber} linked to a statute or legislation?

- No (1)
- Yes (please specify) (2) ________________________________________________
- Unsure (please explain why unsure) (3) ________________________________________________

End of Block: Definitions

Start of Block: Explainer Measurement Tools Loop & Merge

Display This Question:

If Does the article include a measurement of hate crime, hate speech, hate incidents or a surrogate... = Yes

You have indicated that the article contains ${Q2.10/ChoiceTextEntryValue} measurement tool(s). We ask you to code each measurement tool separately. You will be shown the same questions for each measurement tool. Please code each measurement tool individually and make sure that you only code information about the measurement tool in question.

| Page Break |  |
| --- | --- |

End of Block: Explainer Measurement Tools Loop & Merge

Start of Block: Measurement

**You are coding measurement tool # ${lm://CurrentLoopNumber}.**

| Page Break |  |
| --- | --- |

What is the name of the measurement tool #${lm://CurrentLoopNumber}? If more than one measurement tool is described in the document, please insert and code only one at the time. If the tool has no name, leave it blank.

________________________________________________________________

Please specify the author / proponent of measurement tool #${lm://CurrentLoopNumber}'s NAME and AFFILIATION.

________________________________________________________________

Is the measurement tool the operationalisation of a definition of hate crime, hate speech, hate incident or surrogate term?

- Yes (please copy and paste the definition and - if present - the reference) (1) ________________________________________________
- No, the document does not say if the measurement tool refers to any definition (please add if any comment) (2) ________________________________________________

Is the measurement tool #${lm://CurrentLoopNumber} adopted by any governmental or non governmental organisation?

- Not adopted by any organization (9)
- Not specified (8)
- Yes (please specify name of organisation) (1) ________________________________________________

Is measurement tool #${lm://CurrentLoopNumber} at a concept development stage, tested as a pilot, or is it implemented for wide spread application?

- Concept development only (no empirical application) (4)
- Pilot (empirical application but not wide-spread) (1)
- Wide-spread application (2)
- Unsure (please explain why) (3) ________________________________________________

Is measurement tool #${lm://CurrentLoopNumber} freely accessible?

- Yes (please explain) (1) ________________________________________________
- No (please explain) (2) ________________________________________________

What type of measurement tool is #${lm://CurrentLoopNumber}?We define “tool” as is a vehicle or an aid to collect information and data (e.g., an online module to collect data about hate incidents, or an automated text analysis algorithm, or a survey).

- Incident reporting tool (1)
- Survey (2)
- Automated text detection tool (3)
- Other (please specify) (4) ________________________________________________

| Page Break |  |
| --- | --- |

Let's now look at the scope of the measurement tool and what it aims to measure.

What are the target identities that the measurement tool encompasses? Tick all that apply

- Generic (there is no specific identity) (1)
- Racial (please specify if any race is provided) (7) ________________________________________________
- Ethnic (please specify if any ethnicity is provided) (18) ________________________________________________
- National origins (please specify if any nationality is provided) (19) ________________________________________________
- Religious (please specify if any religion is provided) (8) ________________________________________________
- Sexual orientation (please specify if any sexual orientation is provided) (9) ________________________________________________
- People living with a disability (please specify if any disability is provided) (10) ________________________________________________
- Gender (please specify if any gender - including transgender - is provided) (11) ________________________________________________
- Sex (please specify if any sex is provided) (17) ________________________________________________
- Occupation (please specify if any occupation is provided) (12) ________________________________________________
- Age (14)
- Class (15)
- Housing status (16)
- Other (please specify) (13) ________________________________________________

How is the motivation that the measurement tool aims to measure named? Tick all that apply.

- Hate (4)
- Racist (1)
- Antisemitic (2)
- Islamophobic (3)
- Other (please specify) (5) ________________________________________________

How is the behaviour named?

- crime (1)
- incident (2)
- speech (3)
- conduct (4)
- violence (5)
- Other (please specify) (6) ________________________________________________

If the measurement tool provides a list of behaviours that are considered (e.g. murder, vandalism, etc.) please tick all that apply

- The document does not provide a list of behaviours considered by the definitionfo (1)
- murder (2)
- nonnegligent manslaughter (3)
- forcible rape (4)
- robbery (5)
- aggravated assault (6)
- burglary (7)
- theft (8)
- arson (9)
- assault (10)
- intimidation (11)
- destruction / damage / vandalism of property (12)
- other (please specify) (13) ________________________________________________

How is the target of the behavior described?

- Person (1)
- Property (2)
- Organisation (3)
- Group (please specify) (6) ________________________________________________
- Not named (4)
- Other (please specify) (5) ________________________________________________

| Page Break |  |
| --- | --- |

Let's now look at the technical features of the measurement tool.

Display This Question:

If Loop all: What type of measurement tool is #${lm://CurrentLoopNumber}?We define “tool” as is a vehicle or a... = Automated text detection tool

Does the document describe any metrics that the measurement tool adopts to measure hate?

“Metrics” are defined as the parameters (measures) or indices used for measurement, comparison or tracking performance. For example, in relation to automated hate speech detection, they might be vocabularies or lexicon indicating hate speech.

- The document does not contain the description of any metrics (1)
- Parameters for automated hate speech detection (please copy and paste relevant section) (6) ________________________________________________
- Other (please specify) (4) ________________________________________________

Display This Question:

If Loop all: What type of measurement tool is #${lm://CurrentLoopNumber}?We define “tool” as is a vehicle or a... = Survey

Does the document describe any metrics that the measurement tool adopts to measure hate?

“Metrics” are defined as the parameters (measures) or indices used for measurement, comparison or tracking performance. For example, in relation to surveys, they might be bias indicators or the perception of a victim (or a witness) about the bias motivation of the behaviour.

- The document does not contain the description of any metrics (1)
- Bias indicators (please copy and paste relevant section) (6) ________________________________________________
- Perception of a victim or a witness (please copy and paste relevant section) (7) ________________________________________________
- Other (please specify) (4) ________________________________________________

Display This Question:

If Loop all: What type of measurement tool is #${lm://CurrentLoopNumber}?We define “tool” as is a vehicle or a... = Incident reporting tool

Does the document describe any metrics that the measurement tool adopts to measure hate?

“Metrics” are defined as the parameters (measures) or indices used for measurement, comparison or tracking performance. For example, in relation to third-party reporting tools, they might be bias indicators or the perception of a victim (or a witness) about the bias motivation of the behaviour.

- The document does not contain the description of any metrics (1)
- Bias indicators (please copy and paste relevant section) (6) ________________________________________________
- Other (please specify) (4) ________________________________________________

Display This Question:

If Loop all: What type of measurement tool is #${lm://CurrentLoopNumber}?We define “tool” as is a vehicle or a... = Other (please specify)

Does the document describe any metrics that the measurement tool adopts to measure hate?

“Metrics” are defined as the parameters (measures) or indices used for measurement, comparison or tracking performance. For example, in relation to third-party reporting systems or surveys, metrics can be bias indicators. In relation to automated hate speech detection, they might be vocabularies or lexicon indicating hate speech.

- The document does not contain the description of any metrics (1)
- Bias indicators (please copy and paste) (2)
- Other (please specify) (4) ________________________________________________

Does the document discuss feasibility, efficacy or internal validity of the measurement tool #${lm://CurrentLoopNumber}?

- Yes (please copy and paste relevant paragraphs) (1) ________________________________________________
- No (2)
- Unsure (please explain why) (4) ________________________________________________

Are any measurements of validity or reliability of measurement instrument #${lm://CurrentLoopNumber} reported?

- Yes (please copy and paste relevant paragraphs) (1) ________________________________________________
- No (2)
- Unsure (please specify why unsure) (3)

Is there a discussion about whether: 1- measurement tool #${lm://CurrentLoopNumber} captures stability in situations of no change, 2- measurement tool # ${lm://CurrentLoopNumber} detects change in situations of real change

- Yes (please explain) (1) ________________________________________________
- No (please explain) (2) ________________________________________________

Is there a discussion about whether measurement tool #${lm://CurrentLoopNumber} is able to discriminate between groups (e.g. discriminating between hate behaviors and non-hate behaviors)

- Yes (please explain) (1) ________________________________________________
- No (please explain) (2) ________________________________________________

Is there any additional detail about how the measurement tool works? If yes, please copy and paste the relevant sections.

________________________________________________________________

| Page Break |  |
| --- | --- |

Let's now look at the data collected using the measurement tool.

Does the document present the analysis of data collected using measurement tool #${lm://CurrentLoopNumber}?

- Yes (1)
- No (2)
- Unsure (please explain why unsure) (3) ________________________________________________

Display This Question:

If Loop all: Does the document present the analysis of data collected using measurement tool #... = Yes

Is the row data freely accessible?

- Yes (please explain) (1) ________________________________________________
- No (please explain) (2) ________________________________________________

Display This Question:

If Loop all: Does the document present the analysis of data collected using measurement tool #... = Yes

Does the document contain the description of any methods that are used to collect the data?

We define “method” as the process and approach involved in a systematic inquiry of hate crimes, hate incidents, hate speech or surrogate terms, and generally refer to study design or application of an analytical method to this topic. Tick all that apply or type into the "other" section an accurate description of the methods.

- The document does not contain the description of any methods (1)
- Experimental research design (2)
- Cross-sectional quantitative study (3)
- Longitudinal research design (4)
- Qualitative research design (Please specify) (5) ________________________________________________
- Other (please specify) (6) ________________________________________________

Display This Question:

If What type of measurement tool is #${lm://CurrentLoopNumber}?We define “tool” as is a vehicle or a... = Incident reporting tool

Does the incident reporting tool publish data about all incidents that they receive, or only the ones that fall in the ambit of responsibility of the organization? For example, a Human Right Commission that deals with incidents regulated by Civil Law, might receive complaints for incidents regulated by Criminal Law. Are these incidents published as well, or are they deleted from the report?

- All incidents received by the organization, including the ones that fall outside the ambit of responsibility of the organization, are published (1)
- The incidents that are reported to the organization but fall outside its ambit of responsibility are not published (2)
- The information about this is absent from the document (3)
- Unclear - don't know (please specify) (4) ________________________________________________

Display This Question:

If Does the document present the analysis of data collected using measurement tool #... = Yes

Who collected the data? Please provide the name of the organization / institute.

________________________________________________________________

Display This Question:

If Does the document present the analysis of data collected using measurement tool #... = Yes

Please copy and paste a description of the sample composition, data collection method and sample size (for example, how and where the sample was recruited, how the data was collected, who are the respondents)

________________________________________________________________

Display This Question:

If Does the document present the analysis of data collected using measurement tool #... = Yes

What information is provided about the sample? Tick all that apply

- Sample size (please specify) (1) ________________________________________________
- Gender (please specify % of gender groups) (2) ________________________________________________
- Age (please specify % of age groups or mean (SD) (3) ________________________________________________

Display This Question:

If Does the document present the analysis of data collected using measurement tool #... = Yes

And Loop all: What type of measurement tool is #${lm://CurrentLoopNumber}?We define “tool” as is a vehicle or a... = Incident reporting tool

What information about the incident is collected? Please tick all that apply

- The document does not say what information is collected (14)
- Name of reporting person (1)
- Relationship to victim (if reporting person is not the victim) (2)
- Open ended description of the incident (3)
- Date of the incident (4)
- Time of the incident (5)
- Willingness to report the incident to other organizations or services (please specify) (6) ________________________________________________
- Information about the geographical location of the incident (please specify what level, e.g. if postcode, state, other) (7) ________________________________________________
- Contacts of reporting person (8)
- Perceived motivation of the attack (please specify how this is asked) (9) ________________________________________________
- Bias indicators (please specify how they appear) (10) ________________________________________________
- Characteristics of the offender (e.g., age, gender) (please specify) (11) ________________________________________________
- Characteristics of the victim (e.g. age, gender) (please specify) (12) ________________________________________________
- Other (please specify) (13) ________________________________________________

Display This Question:

If Does the document present the analysis of data collected using measurement tool #... = Yes

And Loop all: What type of measurement tool is #${lm://CurrentLoopNumber}?We define “tool” as is a vehicle or a... = Incident reporting tool

What information about the incident is reported? Please tick all that apply

- The document does not report any data (14)
- Name of reporting person (1)
- Relationship to victim (if reporting person is not the victim) (2)
- Open ended description of the incident (3)
- Date of the incident (4)
- Time of the incident (5)
- Willingness to report the incident to other organizations or services (please specify) (6) ________________________________________________
- Information about the geographical location of the incident (please specify what level, e.g. if postcode, state, other) (7) ________________________________________________
- Contacts of reporting person (8)
- Perceived motivation of the attack (please specify how this is asked) (9) ________________________________________________
- Bias indicators (please specify how they appear) (10) ________________________________________________
- Characteristics of the offender (e.g., age, gender) (please specify) (11) ________________________________________________
- Characteristics of the victim (e.g. age, gender) (please specify) (12) ________________________________________________
- Other (please specify) (13) ________________________________________________

End of Block: Measurement
