# Supplementary material for: PROTOCOL: Mapping the scientific knowledge and approaches to defining and measuring hate crime, hate speech, and hate incidents
Source: Campbell Syst Rev. 2022 Apr 18;18(2):e1228. doi: 10.1002/cl2.1228 (PMC9014694; doi:10.1002/cl2.1228)
Supplement: Supplementary file 3 — Supporting information. [file CL2-18-e1228-s002.docx]

ANNEX 3. Search strategies

# Group 1: Databases that allow for complex searches

## EBSCOhost databases

- Communications and Mass Media Complete
- Criminal Justice Abstracts
- SocIndex

| Set# | Search syntax |
| --- | --- |
| S1 | (TI((hate OR prejudice* OR bias* OR racis* OR xenophobi* OR sinophobi* OR anti-foreigner OR anti-migrant* OR anti-immigrant* OR anti-refugee* OR “anti-asylum seeker” OR anti-Roma OR anti-traveller OR anti-Gypsy OR “anti-First Nations” OR anti-Indigenous OR anti-Maori OR anti-Aboriginal OR islamophobi* OR antisemiti* OR anti-semiti* OR anti-Jew* OR anti-Amish OR anti-Sikh OR anti-Buddhis* OR anti-Muslim* OR anti-Islam* OR anti-Christian* OR homophobi* OR transphobi* OR lesbophobi* OR biphobi* OR anti-gay OR anti-lesbian* OR anti-bisex* OR anti-transgender OR anti-LGBT* OR ableis* OR disableis* OR sexis* OR mysogyn* OR misandr* OR gender-based OR “gendered” OR incel OR invcel OR “involuntary celibate” OR anti-feminis* OR anti-abortion OR anti-doctor OR “anti-sex worker” OR anti-politician) N2 ((crim* OR speech OR incident* OR conduct OR act OR abus* OR vilif* OR language* OR violen* OR rape* OR murder* OR harass* OR terroris* OR narrative* OR discourse* OR propaganda OR “targeted violence” OR incite* OR extremis* OR hostil* OR micro-aggression or microaggression OR group*))) OR (AB((hate OR prejudice* OR bias* OR racis* OR xenophobi* OR sinophobi* OR anti-foreigner OR anti-migrant* OR anti-immigrant* OR anti-refugee* OR “anti-asylum seeker” OR anti-Roma OR anti-traveller OR anti-Gypsy OR “anti-First Nations” OR anti-Indigenous OR anti-Maori OR anti-Aboriginal OR islamophobi* OR antisemiti* OR anti-semiti* OR anti-Jew* OR anti-Amish OR anti-Sikh OR anti-Buddhis* OR anti-Muslim* OR anti-Islam* OR anti-Christian* OR homophobi* OR transphobi* OR lesbophobi* OR biphobi* OR anti-gay OR anti-lesbian* OR anti-bisex* OR anti-transgender OR anti-LGBT* OR ableis* OR disableis* OR sexis* OR mysogyn* OR misandr* OR gender-based OR “gendered” OR incel OR invcel OR “involuntary celibate” OR anti-feminis* OR anti-abortion OR anti-doctor OR “anti-sex worker” OR anti-politician) N2 ((crim* OR speech OR incident* OR conduct OR act OR abus* OR vilif* OR language* OR violen* OR rape* OR murder* OR harass* OR terroris* OR narrative* OR discourse* OR propaganda OR “targeted violence” OR incite* OR extremis* OR hostil* OR micro-aggression or microaggression OR group*))) OR (KW((hate OR prejudice* OR bias* OR racis* OR xenophobi* OR sinophobi* OR anti-foreigner OR anti-migrant* OR anti-immigrant* OR anti-refugee* OR “anti-asylum seeker” OR anti-Roma OR anti-traveller OR anti-Gypsy OR “anti-First Nations” OR anti-Indigenous OR anti-Maori OR anti-Aboriginal OR islamophobi* OR antisemiti* OR anti-semiti* OR anti-Jew* OR anti-Amish OR anti-Sikh OR anti-Buddhis* OR anti-Muslim* OR anti-Islam* OR anti-Christian* OR homophobi* OR transphobi* OR lesbophobi* OR biphobi* OR anti-gay OR anti-lesbian* OR anti-bisex* OR anti-transgender OR anti-LGBT* OR ableis* OR disableis* OR sexis* OR mysogyn* OR misandr* OR gender-based OR “gendered” OR incel OR invcel OR “involuntary celibate” OR anti-feminis* OR anti-abortion OR anti-doctor OR “anti-sex worker” OR anti-politician) N2 (crim* OR speech OR incident* OR conduct OR act OR abus* OR vilif* OR language* OR violen* OR rape* OR murder* OR harass* OR terroris* OR narrative* OR discourse* OR propaganda OR “targeted violence” OR incite* OR extremis* OR hostil* OR micro-aggression or microaggression OR group*))) OR (SU((hate OR prejudice* OR bias* OR racis* OR xenophobi* OR sinophobi* OR anti-foreigner OR anti-migrant* OR anti-immigrant* OR anti-refugee* OR “anti-asylum seeker” OR anti-Roma OR anti-traveller OR anti-Gypsy OR “anti-First Nations” OR anti-Indigenous OR anti-Maori OR anti-Aboriginal OR islamophobi* OR antisemiti* OR anti-semiti* OR anti-Jew* OR anti-Amish OR anti-Sikh OR anti-Buddhis* OR anti-Muslim* OR anti-Islam* OR anti-Christian* OR homophobi* OR transphobi* OR lesbophobi* OR biphobi* OR anti-gay OR anti-lesbian* OR anti-bisex* OR anti-transgender OR anti-LGBT* OR ableis* OR disableis* OR sexis* OR mysogyn* OR misandr* OR gender-based OR “gendered” OR incel OR invcel OR “involuntary celibate” OR anti-feminis* OR anti-abortion OR anti-doctor OR “anti-sex worker” OR anti-politician) N2 (crim* OR speech OR incident* OR conduct OR act OR abus* OR vilif* OR language* OR violen* OR rape* OR murder* OR harass* OR terroris* OR narrative* OR discourse* OR propaganda OR “targeted violence” OR incite* OR extremis* OR hostil* OR micro-aggression or microaggression OR group*))) |
| S2 | (TI(“United States” OR “US” OR “USA” OR Australia* OR “New Zealand*” OR Aotearoa OR France OR French OR German* OR Irish OR Ireland OR Ital* OR Spain OR Spanish OR “UK” OR “United Kingdom” OR Brit* OR Engl* OR “Northern Ireland” OR “Northern Irish” OR Scot* OR Wales OR Welsh OR Canad*)) OR (AB(“United States” OR “US” OR “USA” OR Australia* OR “New Zealand*” OR Aotearoa OR France OR French OR German* OR Irish OR Ireland OR Ital* OR Spain OR Spanish OR “UK” OR “United Kingdom” OR Brit* OR Engl* OR “Northern Ireland” OR “Northern Irish” OR Scot* OR Wales OR Welsh OR Canad*)) OR (KW(“United States” OR “US” OR “USA” OR Australia* OR “New Zealand*” OR Aotearoa OR France OR French OR German* OR Irish OR Ireland OR Ital* OR Spain OR Spanish OR “UK” OR “United Kingdom” OR Brit* OR Engl* OR “Northern Ireland” OR “Northern Irish” OR Scot* OR Wales OR Welsh OR Canad*)) OR (SU(“United States” OR “US” OR “USA” OR Australia* OR “New Zealand*” OR Aotearoa OR France OR French OR German* OR Irish OR Ireland OR Ital* OR Spain OR Spanish OR “UK” OR “United Kingdom” OR Brit* OR Engl* OR “Northern Ireland” OR “Northern Irish” OR Scot* OR Wales OR Welsh OR Canad*)) |
| S3 | S1 AND S2 |

## ProQuest databases

- Continental Europe Database
- Dissertations & Theses Global
- ERIC
- Sociological Abstracts
- Technology Collection

| Set# | Search syntax |
| --- | --- |
| S1 | (AB("United States" OR "US" OR "USA" OR Australia* OR "New Zealand*" OR Aotearoa OR France OR French OR German* OR Irish OR Ireland OR Ital* OR Spain OR Spanish OR "UK" OR "United Kingdom" OR Brit* OR Engl* OR "Northern Ireland" OR "Northern Irish" OR Scot* OR Wales OR Welsh OR Canad*) OR TI("United States" OR "US" OR "USA" OR Australia* OR "New Zealand*" OR Aotearoa OR France OR French OR German* OR Irish OR Ireland OR Ital* OR Spain OR Spanish OR "UK" OR "United Kingdom" OR Brit* OR Engl* OR "Northern Ireland" OR "Northern Irish" OR Scot* OR Wales OR Welsh OR Canad*) OR MAINSUBJECT("United States" OR "US" OR "USA" OR Australia* OR "New Zealand*" OR Aotearoa OR France OR French OR German* OR Irish OR Ireland OR Ital* OR Spain OR Spanish OR "UK" OR "United Kingdom" OR Brit* OR Engl* OR "Northern Ireland" OR "Northern Irish" OR Scot* OR Wales OR Welsh OR Canad*)) |
| S2 | (TI(hate OR prejudice* OR bias* OR racis* OR xenophobi* OR sinophobi* OR anti-foreigner* OR anti-migrant* OR anti-immigrant* OR anti-refugee* OR "anti-asylum seeker" OR anti-Roma OR anti-traveller OR anti-Gypsy OR "anti-First Nations" OR anti-Indigenous OR anti-Maori OR anti-Aboriginal OR islamophobi* OR antisemiti* OR anti-semiti* OR anti-Jew* OR anti-Amish OR anti-Sikh OR anti-Buddhis* OR anti-Muslim* OR anti-Islam* OR anti-Christian* OR homophobi* OR transphobi* OR lesbophobi* OR biphobi* OR anti-gay OR anti-lesbian* OR sex* OR anti-transgender OR anti-LGBT* OR ableis* OR disableis* OR sexis* OR mysogyn* OR misandr* OR gender-based OR "gendered" OR incel OR invcel OR "involuntary celibate" OR anti-feminis* OR anti-abortion OR anti-doctor OR "anti-sex worker" OR anti-politician)) NEAR/2 (TI(crim* OR speech OR incident* OR conduct OR act OR abus* OR vilif* OR language* OR violen* OR rape* OR murder* OR harass* OR terroris* OR narrative* OR discourse* OR propaganda OR "targeted violence" OR incite* OR extremis* OR hostil* OR microaggression* OR micro-aggression* OR group*)) |
| S3 | (AB(hate OR prejudice* OR bias* OR racis* OR xenophobi* OR sinophobi* OR anti-foreigner* OR anti-migrant* OR anti-immigrant* OR anti-refugee* OR "anti-asylum seeker" OR anti-Roma OR anti-traveller OR anti-Gypsy OR "anti-First Nations" OR anti-Indigenous OR anti-Maori OR anti-Aboriginal OR islamophobi* OR antisemiti* OR anti-semiti* OR anti-Jew* OR anti-Amish OR anti-Sikh OR anti-Buddhis* OR anti-Muslim* OR anti-Islam* OR anti-Christian* OR homophobi* OR transphobi* OR lesbophobi* OR biphobi* OR anti-gay OR anti-lesbian* OR sex* OR anti-transgender OR anti-LGBT* OR ableis* OR disableis* OR sexis* OR mysogyn* OR misandr* OR gender-based OR "gendered" OR incel OR invcel OR "involuntary celibate" OR anti-feminis* OR anti-abortion OR anti-doctor OR "anti-sex worker" OR anti-politician)) NEAR/2 (AB(crim* OR speech OR incident* OR conduct OR act OR abus* OR vilif* OR language* OR violen* OR rape* OR murder* OR harass* OR terroris* OR narrative* OR discourse* OR propaganda OR "targeted violence" OR incite* OR extremis* OR hostil* OR microaggression* OR micro-aggression* OR group*)) |
| S4 | (MAINSUBJECT(hate OR prejudice* OR bias* OR racis* OR xenophobi* OR sinophobi* OR anti-foreigner* OR anti-migrant* OR anti-immigrant* OR anti-refugee* OR "anti-asylum seeker" OR anti-Roma OR anti-traveller OR anti-Gypsy OR "anti-First Nations" OR anti-Indigenous OR anti-Maori OR anti-Aboriginal OR islamophobi* OR antisemiti* OR anti-semiti* OR anti-Jew* OR anti-Amish OR anti-Sikh OR anti-Buddhis* OR anti-Muslim* OR anti-Islam* OR anti-Christian* OR homophobi* OR transphobi* OR lesbophobi* OR biphobi* OR anti-gay OR anti-lesbian* OR sex* OR anti-transgender OR anti-LGBT* OR ableis* OR disableis* OR sexis* OR mysogyn* OR misandr* OR gender-based OR "gendered" OR incel OR invcel OR "involuntary celibate" OR anti-feminis* OR anti-abortion OR anti-doctor OR "anti-sex worker" OR anti-politician)) NEAR/2 (MAINSUBJECT(crim* OR speech OR incident* OR conduct OR act OR abus* OR vilif* OR language* OR violen* OR rape* OR murder* OR harass* OR terroris* OR narrative* OR discourse* OR propaganda OR "targeted violence" OR incite* OR extremis* OR hostil* OR microaggression* OR micro-aggression* OR group*)) |
| S5 | S1 AND S2 |
| S6 | S1 AND S3 |
| S7 | S1 AND S4 |
| S8 | S5 OR S6 OR S7 |

## Web of Science databases

- Web of Science Core Collection
- SciELO Citation Index

| Set# | Search syntax |
| --- | --- |
| #1 | TS=(hate NEAR/2 (crim* OR speech OR incident* OR conduct OR act OR abus* OR vilif* OR language* OR violen* OR rape* OR murder* OR harass* OR terroris* OR narrative* OR discourse* OR propaganda OR “targeted violence” OR incite* OR extremis* OR hostil* OR micro-aggression or microaggression OR group*)) |
| #2 | TS=(prejudice* NEAR/2 (crim* OR speech OR incident* OR conduct OR act OR abus* OR vilif* OR language* OR violen* OR rape* OR murder* OR harass* OR terroris* OR narrative* OR discourse* OR propaganda OR “targeted violence” OR incite* OR extremis* OR hostil* OR micro-aggression or microaggression OR group*)) |
| #3 | TS=(bias* NEAR/2 (crim* OR speech OR incident* OR conduct OR act OR abus* OR vilif* OR language* OR violen* OR rape* OR murder* OR harass* OR terroris* OR narrative* OR discourse* OR propaganda OR “targeted violence” OR incite* OR extremis* OR hostil* OR micro-aggression or microaggression OR group*)) |
| #4 | TS=(racis* NEAR/2 (crim* OR speech OR incident* OR conduct OR act OR abus* OR vilif* OR language* OR violen* OR rape* OR murder* OR harass* OR terroris* OR narrative* OR discourse* OR propaganda OR “targeted violence” OR incite* OR extremis* OR hostil* OR micro-aggression or microaggression OR group*)) |
| #5 | TS=(xenophobi* NEAR/2 (crim* OR speech OR incident* OR conduct OR act OR abus* OR vilif* OR language* OR violen* OR rape* OR murder* OR harass* OR terroris* OR narrative* OR discourse* OR propaganda OR “targeted violence” OR incite* OR extremis* OR hostil* OR micro-aggression or microaggression OR group*)) |
| … | … |
| #51 | TS=(“anti-sex worker” NEAR/2 (crim* OR speech OR incident* OR conduct OR act OR abus* OR vilif* OR language* OR violen* OR rape* OR murder* OR harass* OR terroris* OR narrative* OR discourse* OR propaganda OR “targeted violence” OR incite* OR extremis* OR hostil* OR micro-aggression or microaggression OR group*)) |
| #52 | TS=(anti-politician NEAR/2 (crim* OR speech OR incident* OR conduct OR act OR abus* OR vilif* OR language* OR violen* OR rape* OR murder* OR harass* OR terroris* OR narrative* OR discourse* OR propaganda OR “targeted violence” OR incite* OR extremis* OR hostil* OR micro-aggression or microaggression OR group*)) |
| #53 | #1 OR #2 OR #3 OR #4 OR #5 … OR #51 OR #52 |
| #54 | TS=(“United States” OR “US” OR “USA” OR Australia* OR “New Zealand*” OR Aotearoa OR France OR French OR German* OR Irish OR Ireland OR Ital* OR Spain OR Spanish OR “UK” OR “United Kingdom” OR Brit* OR Engl* OR “Northern Ireland” OR “Northern Irish” OR Scot* OR Wales OR Welsh OR Canad*) |
| #55 | #53 AND #54 |

## Ovid

- PsycInfo

| Set# | Search syntax |
| --- | --- |
| 1 | ((hate or prejudice* or bias* or racis* or xenophobi* or sinophobi* or anti-foreigner* or  anti-migrant* or anti-immigrant* or anti-refugee* or "anti-asylum seeker" or anti-  Roma or anti-traveller or anti-Gypsy or "anti-First Nations" or anti-Indigenous or  anti-Maori or anti-Aboriginal or islamophobi* or anti-semiti* or anti-Jew* or anti-  Amish or anti-Sikh or anti-Buddhis* or anti-Muslim* or anti-Islam* or anti-Christian*  or homophobi* or transphobi* or lesbophobi* or biphobi* or anti-gay or anti-lesbian*  or anti-bisex* or anti-transgender or anti-LGBT* or ableis* or disableis* or sexis* or  mysogyn* or misandr* or gender-based or "gendered" or incel or invcel or  "involuntary celibate" or anti-feminis* or anti-abortion or anti-doctor or "anti-sex  worker" or anti-politician) adj3 (crim* or speech or incident* or conduct or act or  abus* or vilif* or language* or violen* or rape* or murder* or harass* or terroris* or  narrative* or discourse* or propaganda or "targeted violence" or incite* or extremis*  or hostil* or microaggression* or micro-aggression* or group*)).ab,hw,id,mh,ot,ti. |
| 2 | ("United States" OR "US" OR "USA" OR Australia* OR "New Zealand*" OR Aotearoa OR France OR French OR German* OR Irish OR Ireland OR Ital* OR Spain OR Spanish OR "UK" OR "United Kingdom" OR Brit* OR Engl* OR "Northern Ireland" OR "Northern Irish" OR Scot* OR Wales OR Welsh OR Canad*).ab,hw,id,mh,ot,ti. |
| 3 | 1 and 2 |
| 4 | limit 3 to yr=”1990-2021” |
| 5 | limit 4 to (english or french or german or italian or spanish) |

## Scopus

Search syntax:

TITLE-ABS-KEY(((hate OR prejudice* OR bias* OR racis* OR xenophobi* OR sinophobi* OR anti-foreigner OR anti-migrant* OR anti-immigrant* OR anti-refugee* OR "anti-asylum seeker" OR anti-Roma OR anti-traveller OR anti-Gypsy OR "anti-First Nations" OR anti-Indigenous OR anti-Maori OR anti-Aboriginal OR islamophobi* OR antisemiti* OR anti-semiti* OR anti-Jew* OR anti-Amish OR anti-Sikh OR anti-Buddhis* OR anti-Muslim* OR anti-Islam* OR anti-Christian* OR homophobi* OR transphobi* OR lesbophobi* OR biphobi* OR anti-gay OR anti-lesbian* OR anti-bisex* OR anti-transgender OR anti-LGBT* OR ableis* OR disableis* OR sexis* OR mysogyn* OR misandr* OR gender-based OR "gendered" OR incel OR invcel OR "involuntary celibate" OR anti-feminis* OR anti-abortion OR anti-doctor OR "anti-sex worker" OR anti-politician) W/2 (crim* OR speech OR incident* OR conduct OR act OR abus* OR vilif* OR language* OR violen* OR rape* OR murder* OR harass* OR terroris* OR narrative* OR discourse* OR propaganda OR "targeted violence" OR incite* OR extremis* OR hostil* OR micro-aggression or microaggression OR group*)) AND ("United States" OR "US" OR "USA" OR Australia* OR "New Zealand*" OR Aotearoa OR France OR French OR German* OR Irish OR Ireland OR Ital* OR Spain OR Spanish OR "UK" OR "United Kingdom" OR Brit* OR Engl* OR "Northern Ireland" OR "Northern Irish" OR Scot* OR Wales OR Welsh OR Canad*))

# Group 2: Databases that allow for simple searches

## Science Direct

Each of the search terms below will be combined in a single search using a search string that combines eight search terms using the OR connector. We will search in “Title, abstract or author-specific keywords”. The search string will be as follows.

“hate crime” OR “hate speech” OR “hate incident” OR “hate conduct” OR “hate propaganda” OR “hate group” OR “prejudice-motivated crime” OR “bias crime”

## Google Scholar

In each language, all search terms below will be combined in a single search strings. Parentheses will be used to search for exact phrases, and search terms will be connected using the OR connector (here substituted with |). For each language, we will record the first 200 search results, ordered by relevance.

### English

“hate crime”|“hate speech”|“hate incident”|“hate conduct”|“hate propaganda”|“hate group”|“prejudice-motivated crime”|“bias crime”

### French

“crime de haine”|“discours haineux”|“incident de haine”|“conduite haineuse”|“groupe de haine”|“crime motivé par des préjugés”|“crime biaisé”

### German

“Hasskriminalität”|“Hassrede”|“Hassvorfall”|“Hassverhalten”|“Hassgruppe”|“Hasspropaganda”|“vorurteilsmotivierte Straftat”|“Vorurteilskriminalität”

### Italian

“crimini d’odio”|“discorsi d’odio”|“incidenti di odio”|“comportamento di odio”|“propaganda d’odio”|“gruppi d’odio”| “crimini motivati da pregiudizio”

### Spanish

“crimen de odio”|“discurso de odio”|“incidente de odio”|“conducta de odio”|“propaganda de odio”|“grupo de odio”|“crimen motivado por prejuicio”

## Other databases

We will search each of the databases below using each of the eight search terms individually.

- “hate crime”
- “hate speech”
- “hate incident”
- “hate conduct”
- “hate propaganda”
- “hate group”
- “prejudice-motivated crime”
- “bias crime”

### National Criminal Justice Reference System

We will use the “General Search” search field.

### EU Publications Office

In “Advanced Search”, we will use the search field, enabling the “Exact phrase” option, to search in the “EU Publications” collection.

### EUR-Lex

We will use the “Text search” to search for each search term, enabling the “In Title” and “In Text” checkboxes. We will search in each of the following collections individually

- Treaties
- Legal Acts
- International Agreements
- National Transposition

### Organisation for Security and Economic Co-operation in Europe, Office for Democratic Institutions and Human Rights Document Library

We will use the “Enter your keywords:” fields to search for documents in the “Institutions & Structures” category “OSCE Office for Democratic Institutions and Human Rights”.

### UN Digital Library

We will search in “any field” using the option “All of the words”.

### UN Office of the High Commissioner for Human Rights Digital Library

We will search in “any field” using the option “All of the words”.
