# Supplementary material for: PROTOCOL: Mapping the scientific knowledge and approaches to defining and measuring hate crime, hate speech, and hate incidents
Source: Campbell Syst Rev. 2022 Apr 18;18(2):e1228. doi: 10.1002/cl2.1228 (PMC9014694; doi:10.1002/cl2.1228)
Supplement: Supplementary file 4 — Supporting information. [file CL2-18-e1228-s001.docx]

Grey literature search locations

Country-specific search locations

| Country | Organisation | URL |
| --- | --- | --- |
| Australia | A Gender Agenda | [genderrights.org.au](http://genderrights.org.au/) |
|  | ACON | [acon.org.au](http://acon.org.au/) |
|  | ACT Human Rights Commission | <https://hrc.act.gov.au/> |
|  | ACT Police | [www.police.act.gov.au](http://www.police.act.gov.au/) |
|  | Aged and Disability Advocacy Australia | [adaaustralia.com.au](http://adaaustralia.com.au/) |
|  | Aged Care Quality and Safety Commission | [www.agecarequality.gov.au](http://www.agecarequality.gov.au/) |
|  | Aged Community Services Australia | <https://acsa.asn.au/> |
|  | All Together Now | <https://alltogethernow.org.au/> |
|  | Anglicare Victoria | <https://www.anglicarevic.org.au/> |
|  | Anti-Discrimination NSW | <https://antidiscrimination.nsw.gov.au/> |
|  | Anti-Violence Project Victoria | [antiviolence.info](http://antiviolence.info/) |
|  | Asia Edcuation Australia | [asiaeducation.edu.au](http://asiaeducation.edu.au/) |
|  | Asian Australian Alliance | [asianaustralianalliance.net](http://asianaustralianalliance.net/) |
|  | Asylum Seeker Resource Centre | [asrc.org.au](http://asrc.org.au/) |
|  | Australia/Israel and Jewish Affairs Council | <https://aijac.org.au/> |
|  | Australian Disability Clearinghouse on Education and Training | <https://www.adcet.edu.au/> |
|  | Australian eSafety Commissioner | [esafety.gov.au](http://esafety.gov.au/) |
|  | Australian Federation of Disability Organisations | [afdo.org.au](http://afdo.org.au/) |
|  | Australian Hate Crime Network | <https://www.sydney.edu.au/law/our-research/research-centres-and-institutes/australian-hate-crime-network.html> |
|  | Australian Human Rights Commission | [https://humanrights.org.au](http://humanrights.org.au/) |
|  | Australian Multicultural Foundation | [amf.net.au](http://amf.net.au/) |
|  | Beyond Blue | [beyondblue.org.au](http://beyondblue.org.au/) |
|  | Centre for Multicultural Youth | [cmy.net.au](http://cmy.net.au/) |
|  | Centre for Resilient and Inclusive Societies | [crisconsortium.org](http://crisconsortium.org/) |
|  | Equal Opportunity Commission, Western Australia | <https://www.wa.gov.au/organisation/equal-opportunity-commission> |
|  | Executive Council of Australian Jewry | [www.ecaj.org.au](http://www.ecaj.org.au/) |
|  | Islamic Council of Victoria | [icv.org.au](http://icv.org.au/) |
|  | Islamophobia Register Australia | [islamophobia.com.au](http://islamophobia.com.au/) |
|  | Lowitja Institute | [lowitja.org.au](http://lowitja.org.au/) |
|  | Northern Territory Anti-Discrimination Commission | <https://adc.nt.gov.au/> |
|  | Online Hate Prevention Institute | [ohpi.org.au](http://ohpi.org.au/) |
|  | Our Watch | [ourwatch.org.au](http://ourwatch.org.au/) |
|  | Police Accountability Project | [policeaccountability.org.au](http://policeaccountability.org.au/) |
|  | Redfern Legal Centre | [rlc.org.au](http://rlc.org.au/) |
|  | South Australian Equal Opportunity Commission | [eoc.sa.gov.au](http://eoc.sa.gov.au/) |
|  | Victorian Aboriginal Legal Centre | [www.vals.org.au](http://www.vals.org.au/) |
|  | Victorian Equal Opportunity and Human Rights Commission | [https://www.humanrights.vic.gov.au](http://www.humanrights.vic.gov.au/) |
| Canada | Aboriginal Commission on Human Rights and Justice | <https://www.aboriginalhumanrights.ca/publications> |
|  | Alberta human rights | [https://www.albertahumanrights.ab.ca/](https://www.albertahumanrights.ab.ca/publications/Pages/bulletins_sheets_booklets.aspx) |
|  | Alberta Civil Liberties Research Centre | <https://www.aclrc.com/> |
|  | Amnesty International | [https://www.amnesty.org/en/](https://www.amnesty.org/en/latest/research/page/4/?qlocation=1741&qtopic=2081&qresource-type=2134%2C2146) |
|  | Anti-Defamation League | [https://www.adl.org](https://www.adl.org/) |
|  | B'Nai Brith Canada | [https://www.bnaibrith.ca](https://www.bnaibrith.ca/) |
|  | Canadian Anti-Hate Network | [https://www.antihate.ca](https://www.antihate.ca/) |
|  | Canadian Network for Research on Terrorism, Security and Society | [https://www.tsas.ca](https://www.tsas.ca/) |
|  | Canadian Research Institute for the Advancement of Women (CRIAW-ICREF) | [https://www.criaw-icref.ca/](https://www.criaw-icref.ca/publications/?topic=racism) |
|  | Canadian Women's Foundation | [https://canadianwomen.org/](https://canadianwomen.org/the-facts/) |
|  | Centre for the Prevention of Radicalization Leading to Violence | <https://info-radical.org/en/> |
|  | Coalition of Inclusive Municipalities | <https://en.ccunesco.ca/networks/coalition-of-inclusive-municipalities> |
|  | Commission des droits de la personne et des droits de la jeunesse | [https://www.cdpdj.qc.ca/fr/](https://www.cdpdj.qc.ca/fr/recherche?q=actes+haineux) |
|  | Dawn Canada | [https://dawncanada.net/](https://dawncanada.net/ppbdp-en/reports/) |
|  | Fight Antisemitism | <https://www.fightit.ca/> |
|  | Holy See | <https://www.canadainternational.gc.ca/holy_see-saint_siege/index.aspx?lang=eng> |
|  | Indigenous Bar | <https://indigenousbar.ca/national-secretariat-against-hate-and-racism-in-canada/> |
|  | Justice for Girls | [http://www.justiceforgirls.org/](http://www.justiceforgirls.org/international-human-rights-submissions.html) |
|  | Kantor Centre | [http://www.kantorcenter.tau.ac.il](http://www.kantorcenter.tau.ac.il/) |
|  | Le centre canadien de la diversite des genres + de la sexualite | [https://ccgsd-ccdgs.org/](https://ccgsd-ccdgs.org/research-scholarships/) |
|  | Ligue des droits et libertés, section de Québec | [http://liguedesdroitsqc.org](http://liguedesdroitsqc.org/publications/) |
|  | National Council of Canadian Muslims | [https://www.nccm.ca](https://www.nccm.ca/) |
|  | New Brunswick Human Rights Commission | [https://www2.gnb.ca/content/gnb/en/departments/nbhrc.html](https://www2.gnb.ca/content/gnb/en/departments/nbhrc/resources/annual-reports.html) |
|  | Stats Canada | [https://www.statcan.gc.ca](https://www.statcan.gc.ca/eng/search) |
|  | Strong Voice | [http://www.strongvoice.ca](http://www.strongvoice.ca/) |
|  | The Canadian Council of Muslim Women (CCMW) | [https://www.ccmw.com/](https://www.ccmw.com/publications) |
|  | The Tessellate Institute | [http://tessellateinstitute.com/](http://tessellateinstitute.com/publications/) |
|  | Toronto Police | [http://www.torontopolice.on.ca](http://www.torontopolice.on.ca/publications) |
| France | Commission nationale consultative des droits de l'homme | [https://www.cncdh.fr/](https://www.cncdh.fr/fr/publications) |
|  | Defenseur des Droits | [https://www.defenseurdesdroits.fr/](https://www.defenseurdesdroits.fr/fr/publications?tid=7) |
|  | Délégation Interministérielle à la Lutte Contre le Racisme, l'Antisémitisme et la Haine anti-LGBT (DILCRAH) | [https://www.gouvernement.fr/](https://www.gouvernement.fr/documents-dilcra) |
|  | Haut Conseil à l’égalité entre les femmes et les hommes | <https://www.haut-conseil-egalite.gouv.fr/> |
|  | Institut national d’études démographiques (Ined) | [https://virage.site.ined.fr/](https://virage.site.ined.fr/fr/publications/Publications%20Virage/#r20837) |
|  | Institut des hautes études du ministère de l'Intérieur | [https://www.ihemi.fr](https://www.ihemi.fr/publications?mots_cles%5B%5D=53&sources%5B%5D=12&keyword) |
|  | Ministère de l’Intérieur et Service de Protection de la Communauté Juive | <https://www.antisemitisme.fr/> |
|  | Organization Racism Islamophobia Watch | [https://www.oriw.org/](https://www.oriw.org/category/rapport/page/6/) |
|  | Service Statistique Ministériel de la Sécurité Intérieur (Interstats) | [https://www.interieur.gouv.fr/Interstats/](https://www.interieur.gouv.fr/Interstats/Themes/Menaces-et-injures#84659_children) |
| Germany | Antidiskriminierungsstelle des Bundes | <https://www.antidiskriminierungsstelle.de/DE/startseite/startseite-node.html> |
|  | Bundesministerium der Justiz und für Verbraucherschutz | [https://www.bmjv.de/](https://www.bmjv.de/SharedDocs/Gesetzgebungsverfahren/DE/Bekaempfung_Rechtsextremismus_Hasskriminalitaet.html;jsessionid=B94BB844EB193174205E0B63CCA03509.2_cid334?nn=6704238) |
|  | Bundesverband RIAS | <https://report-antisemitism.de/en/bundesverband-rias> |
|  | Demokratie Zentrum Baden-Württemberg | <https://demokratiezentrum-bw.de/meldestelle-respect/> |
|  | Deutsches Institut für Menschenrechte | <https://www.institut-fuer-menschenrechte.de/themen/rassistische-diskriminierung> |
|  | Hassmelden | <https://hassmelden.de/> |
| Ireland | AkiDwA | [https://akidwa.ie](https://akidwa.ie/) |
|  | Community Work Ireland (CWI) | [https://www.cwi.ie](https://www.cwi.ie/) |
|  | Doras Luimni | <https://doras.org> |
|  | Disability Federation of Ireland | [https://www.disability-federation.ie](https://www.disability-federation.ie/) |
|  | Donegal Intercultural Platform | [https://interculturaldonegal.org](https://interculturaldonegal.org/) |
|  | Inclusion Ireland | [https://inclusionireland.ie](https://inclusionireland.ie/) |
|  | Irish Network Against Racism (INAR) | [https://inar.ie](https://inar.ie/) |
|  | Sex Workers Alliance Ireland | [https://sexworkersallianceireland.org](https://sexworkersallianceireland.org/) |
|  | The Irish Immigrant Support Centre (NASC) | [https://nascireland.org](https://nascireland.org/) |
|  | Transgender Equality Network Ireland (TENI) | [https://www.teni.ie](https://www.teni.ie/) |
|  | Ugly Mugs Ireland | [https://uglymugs.ie](https://uglymugs.ie/) |
| Italy | 1522 (Numero Anti Violenza e Stalking) | https://www.1522.eu/ |
|  | ARCI | [https://www.arci.it/](https://www.arci.it/materiali/) |
|  | Arcigay | <https://www.arcigay.it/> |
|  | Associazione 21 luglio | <https://www.21luglio.org/> |
|  | Centro risorse LGBTI | <https://risorselgbti.eu/> |
|  | Centro Study Sereno Regis | <https://serenoregis.org/> |
|  | CESIE | <https://cesie.org/> |
|  | CIR Rifugiati | <https://www.cir-onlus.org/> |
|  | Donne in rete contro la violenza | <https://www.direcontrolaviolenza.it/> |
|  | Fondazione ISMU | <https://www.ismu.org/en/> |
|  | Gay help line | <https://gayhelpline.it/> |
|  | Ministero Innovazione | <https://innovazione.gov.it/> |
|  | COSPE | [www.cospe.org](http://www.cospe.org/) |
|  | Osservatorio Sicurezza contro gli atti discriminatori (OSCAD) | <https://www.interno.gov.it/it/ministero/osservatori-commissioni-e-centri-coordinamento/osservatorio-sicurezza-contro-atti-discriminatori-oscad> |
|  | Rete Lenford | <https://www.retelenford.it/> |
|  | UNAR | [www.unar.it](http://www.unar.it/) |
| New Zealand | Belong Aotearoa | [beling.org.nz](http://beling.org.nz/) |
|  | Department of Prime Minister and Cabinet | [dpmc.govt.nz](http://dpmc.govt.nz/) |
|  | Foundation Against Islamophobia and Racism | [fairnz.org](http://fairnz.org/) |
|  | Inspector-General of Intelligence and Security | [igis.govt.nz](http://igis.govt.nz/) |
|  | Internet NZ | [internetnz.nz](http://internetnz.nz/) |
|  | Multicultural New Zealand | [multiculturalnz.org.nz](http://multiculturalnz.org.nz/) |
|  | Netsafe | [netsafe.org.nz](http://netsafe.org.nz/) |
|  | New Zealand Indian Central Association | [nzindians.org.nz](http://nzindians.org.nz/) |
|  | New Zealand Security Intelligence Service | [www.nzsis.govt.nz](http://www.nzsis.govt.nz/) |
|  | Office of Ethnic Communities | [ethniccommunities.govt.nz](http://ethniccommunities.govt.nz/) |
|  | Office of Film and Literature Classification | [classificationoffice.govt.nz](http://classificationoffice.govt.nz/) |
|  | Otara Business Association | [otara.co.nz](http://otara.co.nz/) |
|  | Rainbow Mental Health | [rainbowmentalhealth.com](http://rainbowmentalhealth.com/) |
|  | Rainbow Rights | [rainbowrights.nz](http://rainbowrights.nz/) |
|  | Rainbow Youth | [ry.org.nz](http://ry.org.nz/) |
|  | Royal Commission of inquiry into the Attack on Christchurch Mosques on 15 March 2019 | <https://christchurchattack.royalcommission.nz/> |
|  | Shine | [2shine.org.nz](http://2shine.org.nz/) |
|  | The Federation of Islamic Associations of New Zealand (Inc) | [fianz.com](http://fianz.com/) |
|  | The Helen Clark Foundation | https://helenclark.foundation/ |
|  | Third Culture Minds | [thirdcultureminds.org.nz](http://thirdcultureminds.org.nz/) |
|  | Umma Trust | [ummatrust.co.nz](http://ummatrust.co.nz/) |
|  | Women's Refuge | [womensrefuge.org.nz](http://womensrefuge.org.nz/) |
|  | Working Together Group | [wtg.org.nz](http://wtg.org.nz/) |
| Spain | Accem | <https://www.accem.es/> |
|  | Asociación Musulmana por los Derechos Humanos (AMDEH) | <https://amderechoshumanos.org/> |
|  | Ministerio del Interior, Servicios al Ciudadano, Delitos de Odio | <http://www.interior.gob.es/es/web/servicios-al-ciudadano/delitos-de-odio> |
|  | Ministerio Fiscal | <https://www.fiscal.es/web/fiscal/-/delitos-de-odio-y-discriminacion> |
|  | Movimiento contra la Intolerancia | <http://movimientocontralaintolerancia.com/> |
|  | Observatorio Español del Racismo y la Xenofobia | <https://www.inclusion.gob.es/oberaxe/es/index.htm> |
|  | Plataforma Khetane | <https://plataformakhetane.org/> |
|  | Union de Comunidades Islámicas en España | <https://ucide.org/> |
| UK | Crown Prosecution Service | [https://www.cps.gov.uk](https://www.cps.gov.uk/) |
|  | Equality and Human Rights Commission | <https://www.equalityhumanrights.com/en> |
|  | INACH | <https://www.inach.net/united-kingdom/> |
|  | International Network for Hate Studies | [https://internationalhatestudies.com](https://internationalhatestudies.com/) |
|  | Opinium | <https://www.opinium.com/multicultural-britain/> |
|  | Runny Mede | [https://www.runnymedetrust.org/](https://www.runnymedetrust.org/uploads/Islamophobia%20Report%202018%20FINAL.pdf) |
|  | Stop Hate UK | [https://www.stophateuk.org/](https://www.stophateuk.org/wp-content/uploads/2010/07/Stop-Hate-UK-Stats-Report-2015-16-Final.pdf) |
|  | TellMAMA | [https://tellmamauk.org/](https://tellmamauk.org/wp-content/uploads/resources/Tell%20MAMA%20-%20Report.pdf) |
|  | Law Commission | [https://www.lawcom.gov.uk](https://www.lawcom.gov.uk/) |
|  | True Vision | <https://www.report-it.org.uk/> |
|  | Commission for Countering Extremism (CCE) | <https://www.gov.uk/government/organisations/commission-for-countering-extremism> |
|  | Centre for Hate Studies, University of Leichester | <https://le.ac.uk/hate-studies> |
| US | Anti-Defamation League | [https://www.adl.org](https://www.adl.org/) |

Supra-national / international organisations

| Name of supra-national / international organisation | Name of Organisation | URL |
| --- | --- | --- |
| EU | Eurobarometer | <https://europa.eu/eurobarometer/> |
|  | Eurostat | ec.europa.eu/eurostat |
|  | European Commission against Racism and Intolerance (ECRI) | <https://www.coe.int/en/web/european-commission-against-racism-and-intolerance> |
|  | ECRI – Human Rights Documentation | https://hudoc.ecri.coe.int/eng |
|  | Fundamental Rights Agency | [https://fra.europa.eu/en](https://fra.europa.eu/en/promising-practices-list) |
| OSCE | Office for Democratic Institutions and Human Rights | https://www.osce.org/odihr |
| UN | Special rapporteur on contemporary forms of racism, Office of the High Commissioner for Human Rights | https://www.ohchr.org/EN/Issues/Racism/SRRacism/Pages/IndexSRRacism.aspx |

International Initiatives

| Name of initiative | URL |
| --- | --- |
| Christchurch Call | <https://www.christchurchcall.com/> |
| Global Internet Forum to Counter Terrorism | https://gifct.org/ |

Tech Companies

| Name of company | URL |
| --- | --- |
| YouTube | https://www.youtube.com |
| *Twitter* | https://twitter.com |
| *Microsoft* | https://www.microsoft.com |
| *facebook* | https://www.facebook.com |
| *tumblr* | https://www.tumblr.com |
| *WordPress.com* | https://www.wordpress.com |
| *JustPaste.it* | https://justpaste.it/ |
| *Airbnb* | https://www.airbnb.com |
| *mailchimp* | https://mailchimp.com/ |
| *Discord* | https://discord.com/ |
| *Instagram* | https://www.instagram.com |
| *WhatsApp* | https://www.whatsapp.com |
| *Pinterest* | https://www.pinterest.com |
| *Amazon* | https://www.amazon.com |
| *Dropbox* | https://www.dropbox.com |
| *MEGA* | https://mega.io/ |
| *Linkedin* | https://www.linkedin.com |
